# Combined transcriptome and metabolome analyses of metformin effects reveal novel links between metabolic networks in steroidogenic systems

**DOI:** 10.1038/s41598-017-09189-y

**Published:** 2017-08-17

**Authors:** Sameer S. Udhane, Balazs Legeza, Nesa Marti, Damian Hertig, Gaëlle Diserens, Jean-Marc Nuoffer, Peter Vermathen, Christa E. Flück

**Affiliations:** 1 Pediatric Endocrinology and Diabetology of the Department of Pediatrics and the Department of Clinical Research, University of Bern, 3010 Bern, Switzerland; 2 Departments of Clinical Research and Radiology, University of Bern, Bern, Switzerland; 3 University Institute of Clinical Chemistry, University of Bern, Bern, Switzerland

## Abstract

Metformin is an antidiabetic drug, which inhibits mitochondrial respiratory-chain-complex I and thereby seems to affect the cellular metabolism in many ways. It is also used for the treatment of the polycystic ovary syndrome (PCOS), the most common endocrine disorder in women. In addition, metformin possesses antineoplastic properties. Although metformin promotes insulin-sensitivity and ameliorates reproductive abnormalities in PCOS, its exact mechanisms of action remain elusive. Therefore, we studied the transcriptome and the metabolome of metformin in human adrenal H295R cells. Microarray analysis revealed changes in 693 genes after metformin treatment. Using high resolution magic angle spinning nuclear magnetic resonance spectroscopy (HR-MAS-NMR), we determined 38 intracellular metabolites. With bioinformatic tools we created an integrated pathway analysis to understand different intracellular processes targeted by metformin. Combined metabolomics and transcriptomics data analysis showed that metformin affects a broad range of cellular processes centered on the mitochondrium. Data confirmed several known effects of metformin on glucose and androgen metabolism, which had been identified in clinical and basic studies previously. But more importantly, novel links between the energy metabolism, sex steroid biosynthesis, the cell cycle and the immune system were identified. These omics studies shed light on a complex interplay between metabolic pathways in steroidogenic systems.

## Introduction

Androgens are crucial steroid hormones for normal sexual development and reproduction in males and females. Main androgen producing steroid tissues are the fetal adrenals as well as the adult zona reticularis (ZR) of the adrenal cortex, and the male and female gonads^1^. Androgens are synthesized from cholesterol, which serves as essential substrate for the production of pregnenolone in the mitochondria. Further conversion of pregnenolone to androgens requires the presence of specific steroid enzymes and cofactors in a catalytic cascade located in the endoplasmatic reticulum (ER) and the mitochondria. Two enzymes are essential for androgen production, namely CYP17 (P450c17, encoded by *CYP17A1*) and type 2 3β-hydroxysteroid dehydrogenase (HSD3B2/3βHSDII, encoded by *HSD3B2*); without their enzyme activities androgen production is lost^1^.

In humans, two common hyperandrogenic conditions, premature adrenarche and the polycystic ovary syndrome (PCOS), are still poorly understood. Adrenarche is a physiologic event almost unique to humans around age 8 years, when the ZR starts to produce adrenal androgens. Adrenarche is characterized by lower HSD3B2 activity and increased CYP17-lyase activity for higher androgen production^2–4^. However, the function and the regulators that trigger adrenarche are still unknown. Accordingly, the meaning of premature adrenarche remains elusive. Some studies suggest that girls who had premature adrenarche are more likely to develop PCOS^5, 6^. PCOS is a common, heritable, complex disorder characterized by hyperandrogenism (clinical or biochemical), chronic oligo- or anovulation and polycystic ovaries (PCO)^7^. Besides reproductive consequences, PCOS has also very often important metabolic consequences including insulin resistance bearing an increased risk for type 2 diabetes^8^. Current treatment options of PCOS are scarce and largely symptomatic due to lack of knowledge of its underlying pathomechanisms.

The biguanide analog metformin has been used to treat type 2 diabetes (T2DM) for decades. Besides its inhibitory effects on hepatic glucose production, it increases glucose uptake in peripheral tissues and reduces fatty acid oxidation^9, 10^. Since insulin resistance plays an important role in the pathogenesis of PCOS, insulin-sensitizing drugs such as metformin were also used in diabetic women with PCOS. In the first report, metformin did not only improve diabetes, but also ameliorated reproductive abnormalities; it restored ovulation and reduced androgenic symptoms^11^. Therefore, metformin was widely adopted as therapy for PCOS^12^, although its mechanism of action on androgen production was unknown.

Unlike effects on androgen production, metformin’s action on glucose metabolism has been extensively studied. Metfomin decreases cellular respiration by inhibiting specifically complex I (NADH:ubiquinone oxidoreductase) of the respiratory-chain without affecting other complexes of the mitochondrial respiratory chain^13^. In general, inhibition of complex I decreases the cellular ATP concentration and diminishes cellular energy^14^. Increased ADP/ATP and AMP/ATP ratios then activate the AMP-activated protein kinase (AMPK), a critical cellular energy sensor that integrates multiple metabolic signaling networks^15^. Additional effects of the metformin altered AMP/ATP ratio include an improved lipid metabolism by suppressing hepatic lipid synthesis^16^ and an increased fatty acid oxidation through the AMPK-dependent phosphorylation of acetyl CoA carboxylase (ACC)^17^. Furthermore, AMPK-independent, direct AMP- and ATP-mediated effects of metformin are reported to modulate gluconeogenesis. Increased levels of AMP exert a direct allosteric effect on fructose 1,6-bisphosphatase^18^, and inhibit adenylate cyclase and cyclic AMP (cAMP)-protein kinase A (PKA) signaling in response to glucagon^19^, which both lead to reduced gluconeogenesis. In addition, metformin also inhibits mitochondrial glycerophosphate dehydrogenase (mGPD), which results in reduced conversion of glycerol to glucose and an altered redox state; enhanced cytosolic NADH then feeds back on lactate dehydrogenase activity^20^.

Similarly, metformin reduces androgen production of steroidogenic H295R cells through inhibition of complex I (Supplementary Figure 1)^21^. In previous studies, we showed that human adrenal H295R cells shift their steroid profile and produce more androgens under starvation growth conditions^22–26^. In this condition activities of HSD3B2 and CYP17 were changed as typically seen with adrenarche^23, 24^, thus providing an *in vitro* model for further studies of androgen regulation. Therefore, this H295R cell model was used in starvation (hyperandrogenic) and metformin treated (hypoandrogenic) conditions to search for the underlying androgen regulating network and obtain further insight into basic androgen biology. Transcriptome analysis revealed 14 differentially expressed genes involved in steroid biosynthetic processes (HSD3B1, HSD3B2 and CYP21A2), energy metabolism and signal transduction (retinoic acid receptor beta (RARB) and angiopoietin-like protein 1 (ANGPTL1)^26, 27^. These studies were now extended for metformin effects. In addition, metabolic profiling studies using NMR spectroscopy were performed to also assess effects at the metabolic level. Taking a systems biology approach, all data were then subjected to integrated network analyses looking for common regulatory networks involved in mitochondrial metabolism, steroidogenesis and PCOS disease state. Having performed all these studies we are now able to provide a detailed map of metformin targeted genes, proteins and metabolites in an androgen producing cell system.

## Results

### Characterization of the gene expression profile of human adrenal H295R cells under metformin treatment

**Figure 1:**
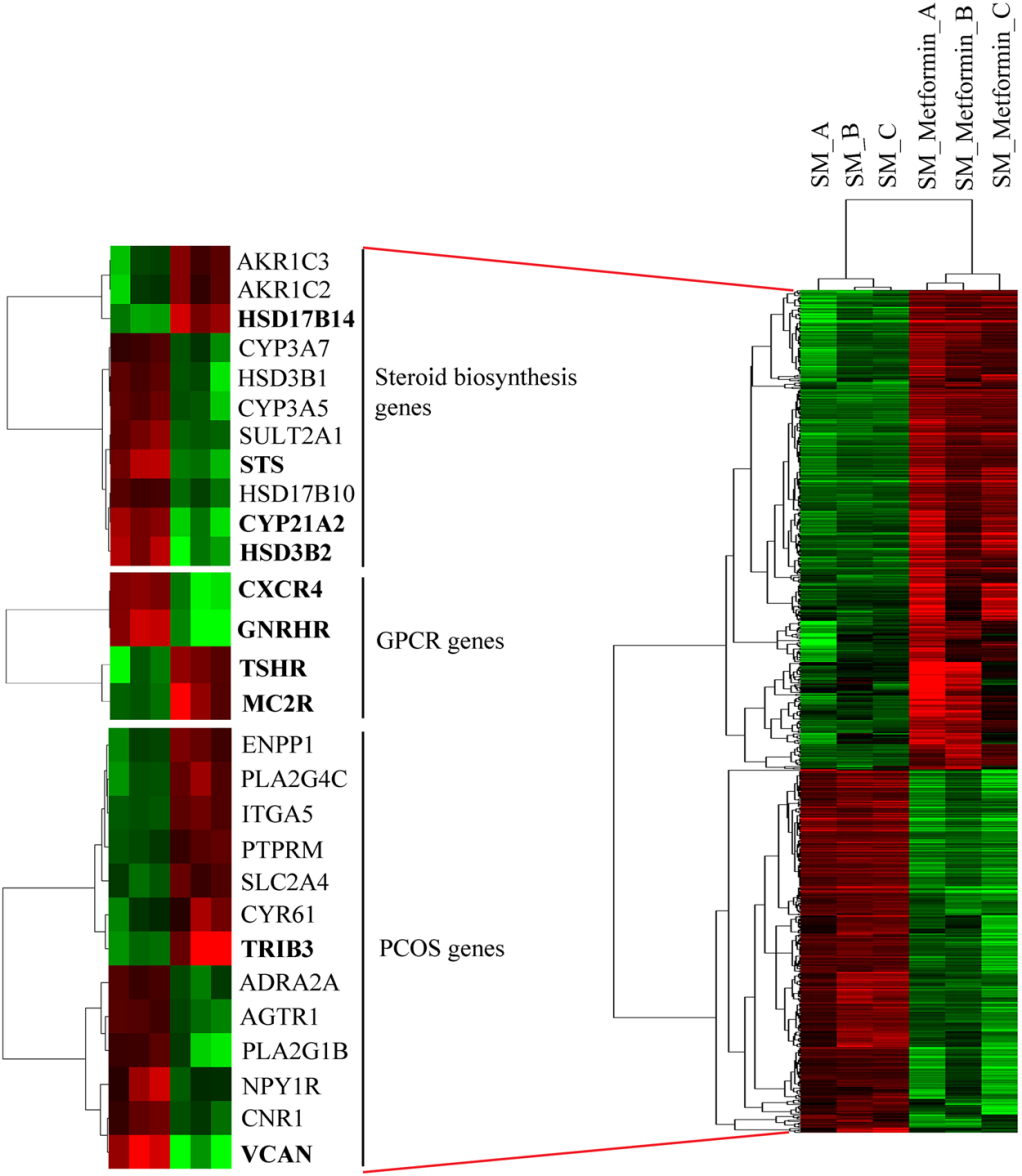
Heat map of Affymetrix microarray data showing differentially expressed genes in H295R cells grown under serum starvation (SM) conditions with and without metformin treatment. Microarray data were analyzed by Cluster 3.0 and JTreeview software to generate a representative heat map. The heat map on the right side shows 693 genes, which were found differentially expressed when testing for a fold change level of gene expression set at >1.5. Genes highlighted in bold were already identified at a level of >2.0 fold change in gene expression. The heat map on the left shows identified genes specifically involved in steroid biosynthetic processes, G-protein coupled receptor (GPCR) biology, and genes associated with polycystic ovary syndrome (PCOS), a hyperandrogenic disease condition. In the heat map graphics, rows show individual genes. Triplicate samples are depicted in columns. Gene expression levels are displayed for each independent sample. Over-expression is shown in red, under-expression in green.

Microarray studies on starved H295R cells treated with metformin for 48 hours were performed using GeneChip Human Gene 1.0 ST arrays. A total of 693 genes were found altered in their expression (>1.5 fold change; n of 104 at >2.0 fold change (Supplementary Table 1)) after metformin treatment, of which 398 were up-regulated and 295 were down-regulated. Data were further analyzed for hierarchical clustering using the complete linkage algorithm of Cluster 3.0 and a heat map was created for visualization of the data by JTreeView (Fig. 1). The identified genes were subjected to enrichment analysis to rank for enriched biological processes/networks (Table 1) and for diseases biomarkers (Supplementary Table 2). Interestingly, these analyses revealed the involvement of several genes important for steroidogenesis and PCOS that were altered by metformin treatment, e.g. genes of steroid biosynthesis (HSD17B14, STS, CYP21A2, HSD3B2), GPCR genes (CXCR4, GnRHR, TSHR, MC2R) and PCOS genes (TRIB3, VCAN, ENPP1, ITGA5, PTPRM, SLC2A4, CYR61, ADRA2A, AGTR1, NPY1R and CNR1).

**Table 1:** Enrichment analysis of differentially expressed genes in H295R cells under starvation conditions vs metformin treatment.

Enrichment Analysis		SM vs Met			
	Pathway Maps	p-value	FDR	n	Genes
1	Cell Cycle_start of DNA replication in early S phase	0.00006	0.03	6	Histone H1, CDC45L, MCM2, MCM10, ORC1L, MCM3
2	Androstenedione and testosterone biosynthesis	0.00097	0.26472	6	HSD3B1, CYP3A7, CYP3A5, SULT2A1, **HSD3B2**, **STS**
	**GO Processes**				
1	Chromatin assembly or disassembly	≤ 0.00001	≤ 0.00001	30	Histone H1, Histone H1, HIST1H3D, Histone H3, HIST1H2AE, HIST1H2BA, C/EBPgamma, C/EBP, MCM2, Histone H2, Histone H2A, Histone H4, Histone H2B, HIST1H2BN, HIST2H2AC, CINAP, HIST1H2BG, HIST1H2AD, Histone H2A.o, Histone H2BO, HIST1H2AH, TSPYL5, CENP-50, BAF57, Histone H1.5, HIST1H2BM, HIST2H2BE, HIST1H2BB, Histone deacetylase class I, HIST1H2BJ
2	G-protein coupled receptor signaling pathway, coupled to cyclic nucleotide second messenger	≤ 0.00001	0.00003	22	G-protein gamma, CCL2, Galpha(s)-specific hormone protein GPCRs, TSH receptor, Galpha(i)-specific amine GPCRs, Alpha-2A adrenergic receptor, Galpha(i)-specific peptide GPCRs, Galpha(q)-specific peptide GPCRs, NPY1R, CACNA1 L-type, PDE, Galpha(s)-specific peptide GPCRs, **MC2R**, Galpha(i)-specific metabotropic glutamate GPCRs, mGluR7, CNR1, Galpha(i)-specific cannabis GPSRs, PDE4, PDE4D, G-protein gamma 2, Kainate receptor, Ionotropic glutamate receptor
3	Mineralocorticoid biosynthetic process	≤ 0.00001	0.0001	5	HSD3B1, CACNA1H, CACNA1 T-type, **HSD3B2**, **CYP21A2**
4	Parturition	≤ 0.00001	0.00009	8	PLA2G4C, PLA2, cPLA2, CCL2, Galpha(q)-specific peptide GPCRs, DAF, TK1, AKR1C3
	**Process Networks**				
1	Cell cycle S phase	0.00003	0.005480	16	Stromalins 1/2, Histone H1, CDC45L, RFC1, MCM2, p21, Histone H4, AHR, MCM10, MCM7, ORC1L, Histone H1.5, POLA1, MCM3, DNA ligase I, BRIP1
2	Cell cycle_Core	0.0004	0.003	12	CDC45L, CDC25A, MCM2, p21, Cyclin E2, ZW10, MCM10, MCM7, ORC1L, MCM3, DNA ligase I, CAP-G

Differentially expressed genes in serum starvation condition (SM) vs metformin (Met) treatment were analyzed with the GeneGo Metacore software to obtain an enrichment analysis. Analysis was performed on the 693 gene transcripts identified by microarray analysis setting the fold change cut-off at 1.5 with an adjusted p-value < 0.05. Genes given in bold were identified at a cut-off 2.0-fold. Results of the enrichment analysis for significant Pathway Maps, GO Processes and Process Networks are shown. Please note that in the Pathway maps, androstenedione and testosterone biosynthesis were non-significant pathways, but they are nevertheless included for their relevance to our study. (FDR = false discovery rate; n = number of genes identified).

**Figure 2:**
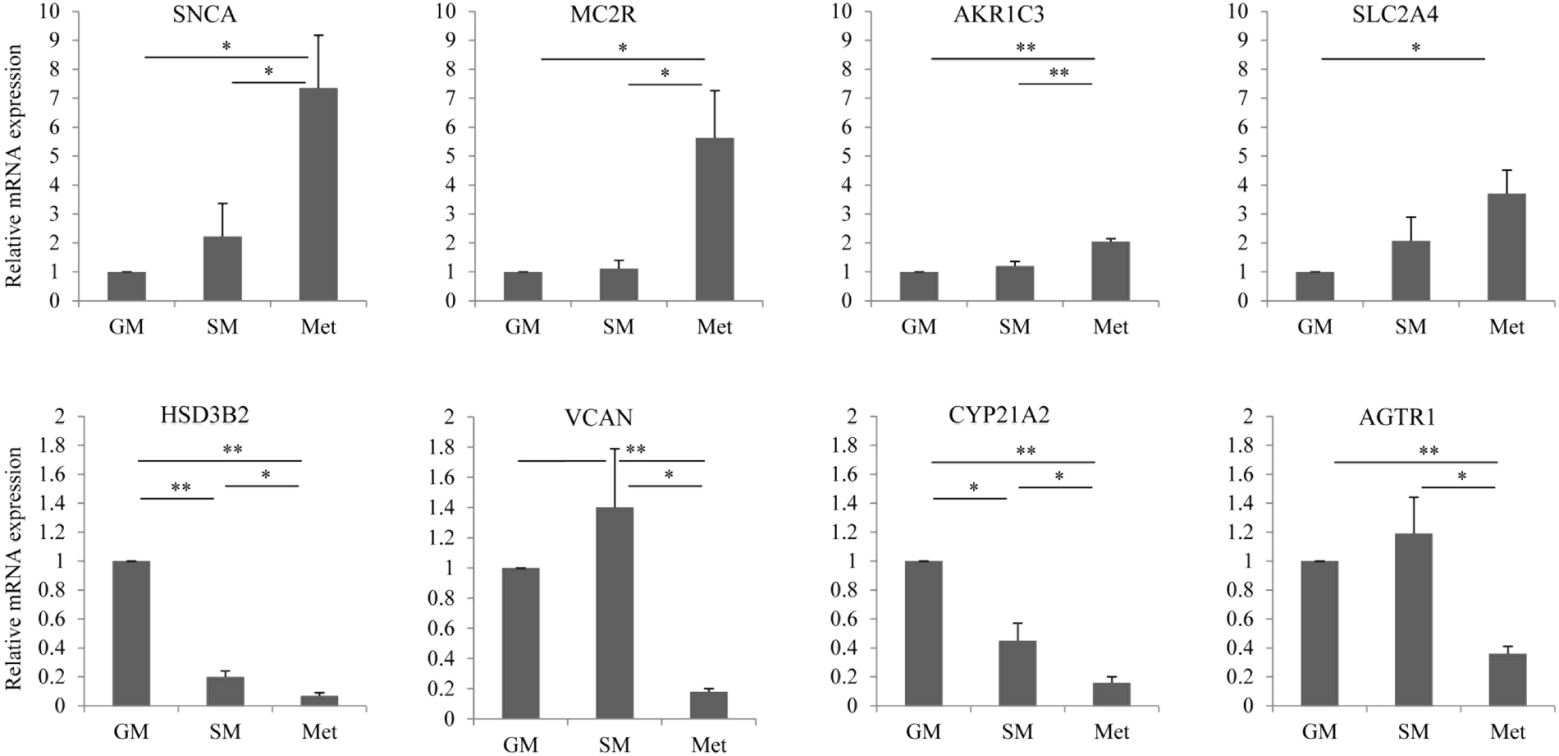
Validation of gene expression data obtained from microarray analysis by qRT-PCR. Confirmatory gene expression profiling was performed using same total RNA from H295R cells grown under starvation conditions (SM) without and with metformin (Met) treatment. Eight genes identified by microarrays with a significance level of fold change >1.5 (4 of them, namely MC2R, HSD3B2, VCAN and CYP21A2 more than 2.0 fold) were selected for validation and analyzed by SYBER Green based qRT-PCR. Analysis of relative gene expression was performed according to the 2^−ΔΔCt^ method using GAPDH for normalization. Results are presented as mean ± SD of three independent experiments. *p < 0.05, **p < 0.01. GM – growth medium.

To validate our microarray data, we selected eight representative genes with a significant up- or down-regulation of more than 1.5 fold change (p < 0.05) under metformin treatment, and performed qRT-PCR experiments. Metformin up-regulated genes tested were SNCA, MC2R, AKR1C3 and SLC2A4 (also known as GLUT4), and were all confirmed for significantly higher expression in metformin treated H295R cells, when compared to normal and starvation gowth conditions (Fig. 2, upper panel). Metformin down-regulated genes HSD3B2, VCAN, CYP21A2 and AGTR1, identified by microarrays, were also validated by qRT-PCR, and were all confirmed for significantly lower expression (Fig. 2, lower panel). Of note, qRT-PCR results also confirmed significantly lower expression of HSD3B2 and CYP21A2 under starvation compared to normal growth conditions as described in previous microarray studies^26^.

### Network architecture of genes modulated by metformin in H295R cells

**Figure 3:**
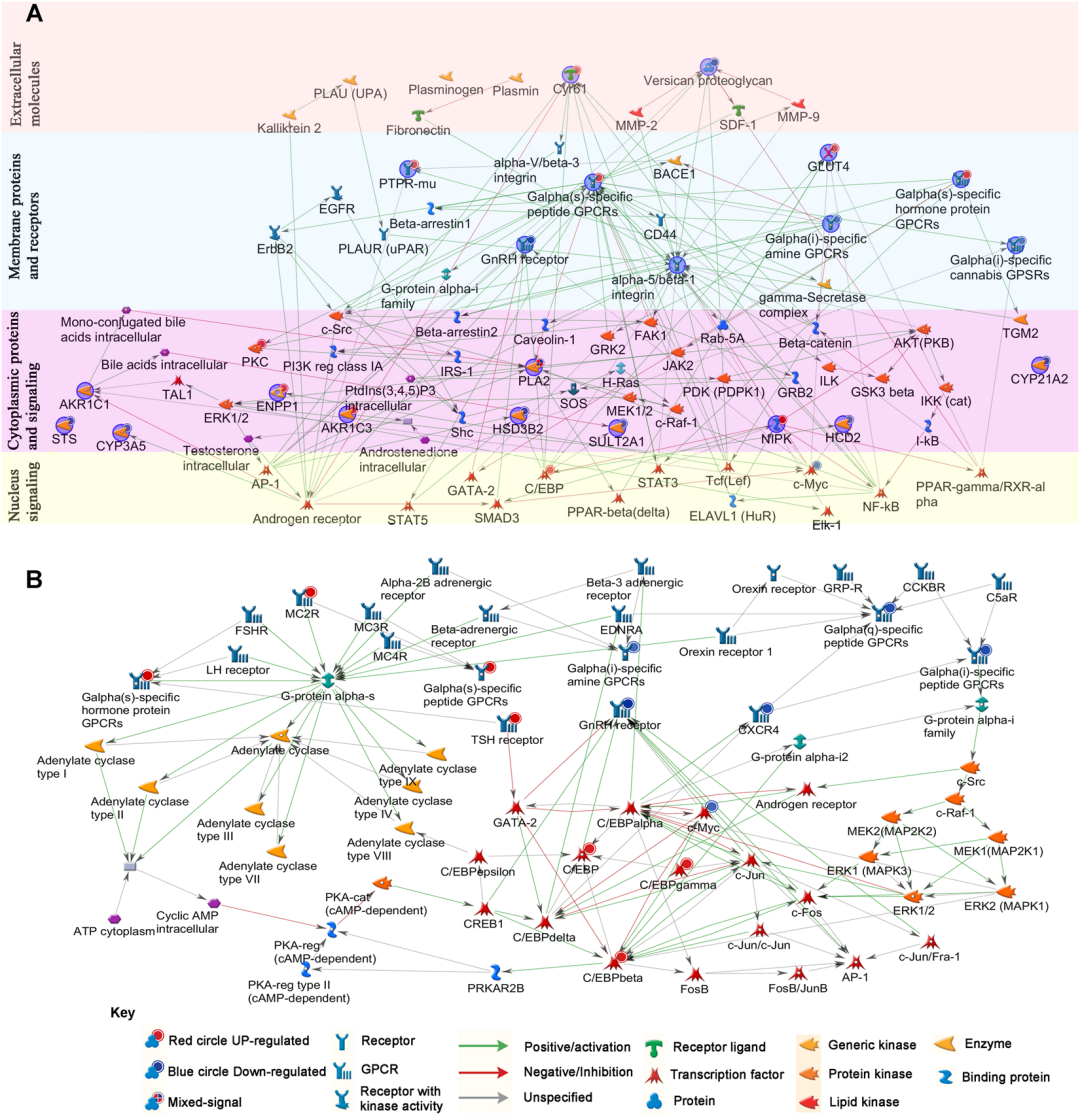
Gene network targeted by metformin treatment in H295R cells when analyzing for steroid biosynthetic processes, GPCRs and PCOS. Using the auto expand algorithm from the GeneGo Metacore software, we build the connecting networks between the differentially expressed genes of interest. We identified multiple gene networks that are involved in modulating both intra- and extracellular proteins. (**A**) Overview of the identified gene network according to the localization of the encoded proteins. Four color represent different localization of the encoded proteins, enzymes, signaling pathways. Nodes circled in blue color show the genes of interest found in our microarray data. Up-regulated genes are marked with small superscript red circles, while down-regulated genes are marked with small superscript blue circles. Green lines show activating relationships, red lines show an inhibition; gray lines show relationships of unknown quality. (**B**) Network analysis of differentially expressed GPCR genes targeted by metformin treatment. Cyclic AMP and MAPK signaling pathways, which are known regulators of androgen production, were highlighted by our analysis. Up- and down-regulated genes found in this network are marked with small superscript red circles for up-regulated genes and small superscript blue circle for down-regulated genes. Green lines show activating relationships; red lines show an inhibition; gray lines show relationships of unknown quality. Further classification of the proteins is given in the key section of the figure.

Metformin has been reported to alter cellular mechanisms in a complex, yet not fully elucidated manner^14, 28^. We therefore performed network analysis for metformin effects, applying the auto-expand algorithm on selected genes related to PCOS and intracellular signaling. This analysis revealed a complex network between genes for GPCRs, steroid biosynthesis and PCOS (Fig. 3). Metformin seems to target membrane proteins and extracellular as well as intracellular protein signaling networks, of which many are known to be involved in androgen production (Fig. 3A). This network comprises extracellular proteins (CYR61 and VCAN), membrane receptors (GPCRs, GnRH receptor), cytoplasmic proteins and signaling cascades (SDRs, CYP enzymes, as well as PLA2 and PKC pathway), and several transcription factors (C/EBPs, STAT5, GATA2). Interestingly, an interrelationship between the androgen receptor and cytochrome P450 (CYP) and SDR enzymes (AKR1C3, AKR1C2 and SULT2A1) was detected. In addition, a direct link between C/EBP family of transcription factors and HSD3B2 was found on the metformin regulated gene map. In line with our results, C/EBPs have just recently been shown to regulate HSD3B2, CYP11A1 and StAR expression in steroidogenic granulosa tumor-derived KGN cells^29^.

Because metformin altered the expression of several GPCR genes (e.g. MC2R and GnRHR), which are essential for the regulation of androgen production, we also performed network analysis for GPCR genes (Fig. 3B). This analysis revealed that GPCRs connect downstream to cAMP and MAPK signaling pathways, which control CYP17A1 and HSD3B2 expression and thus androgen production^23, 25, 27, 30^. The network analysis also suggested that metformin targeted cAMP and MAPK signaling alters downstream transcription factors C/EBP gamma and beta, which may play a role in endometrial receptivity^31, 32^.

### Comparison of the gene expression profiles of starved (hyperandrogenic) and metformin treated (androgen inhibited) H295R cells

**Figure 4:**
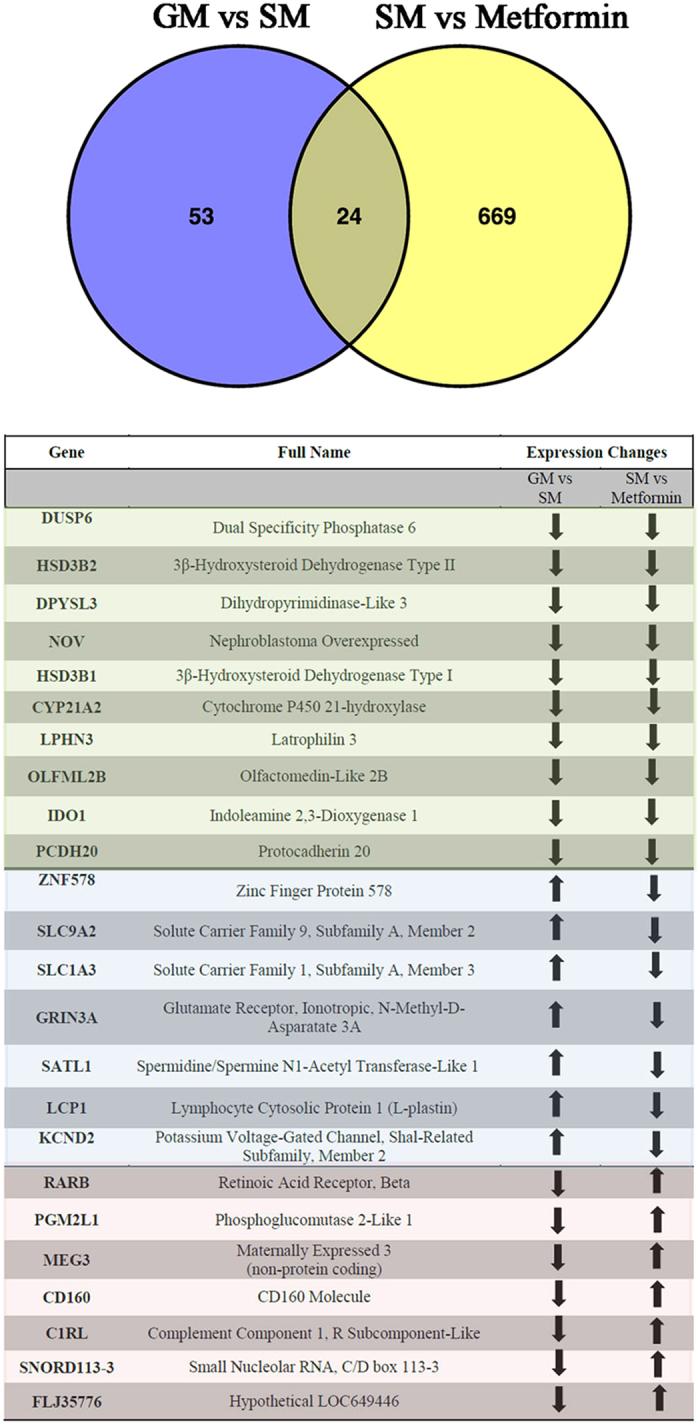
Metformin regulates 693 genes, of which 24 are also regulated by starvation growth conditions. Comparison of differentially expressed genes (>1.5 fold) in human H295R cells grown under normal (GM) and starvation (SM) growth conditions and under metformin (Met) treatment was performed. For GM vs SM condition, 77 genes were differentially expressed^26^. For SM vs Met, 693 genes were significantly altered (this study). Comparison between the two groups revealed 24 genes that were regulated in both; expression of 10 genes was repressed in both (green color), 14 genes were altered in opposite directions between the two groups (labelled with blue and red color). Exact names and the expression pattern of the 24 genes regulated by both starvation and metformin are given in tabulated form.

The transcriptome of H295R cells was assessed in the hyperandrogenic versus androgen inhibited state. Common alterations were found in a total of 24 genes (>1.5-fold, p < 0.05) (Fig. 4). Of this 24 common genes, 14 genes were regulated in opposite direction, while 10 were down-regulated in both conditions. For example gene expression levels of HSD3B1, HSD3B2, CYP21A2, DPYSL3, IDO1 and NOV were downregulated in both conditions; by contrast, retinoic acid receptor beta (RARB) was down- in starvation, but up-regulated with metformin treatment (Fig. 4, list). RARB has been shown to regulate androgen production through StAR, HSD3B2/Nur77 and CYP17A1 in starvation^26^.

### Effects of metformin on the intracellular metabolism of H295R cells revealed by transcriptome analysis and by NMR spectroscopy

**Figure 5:**
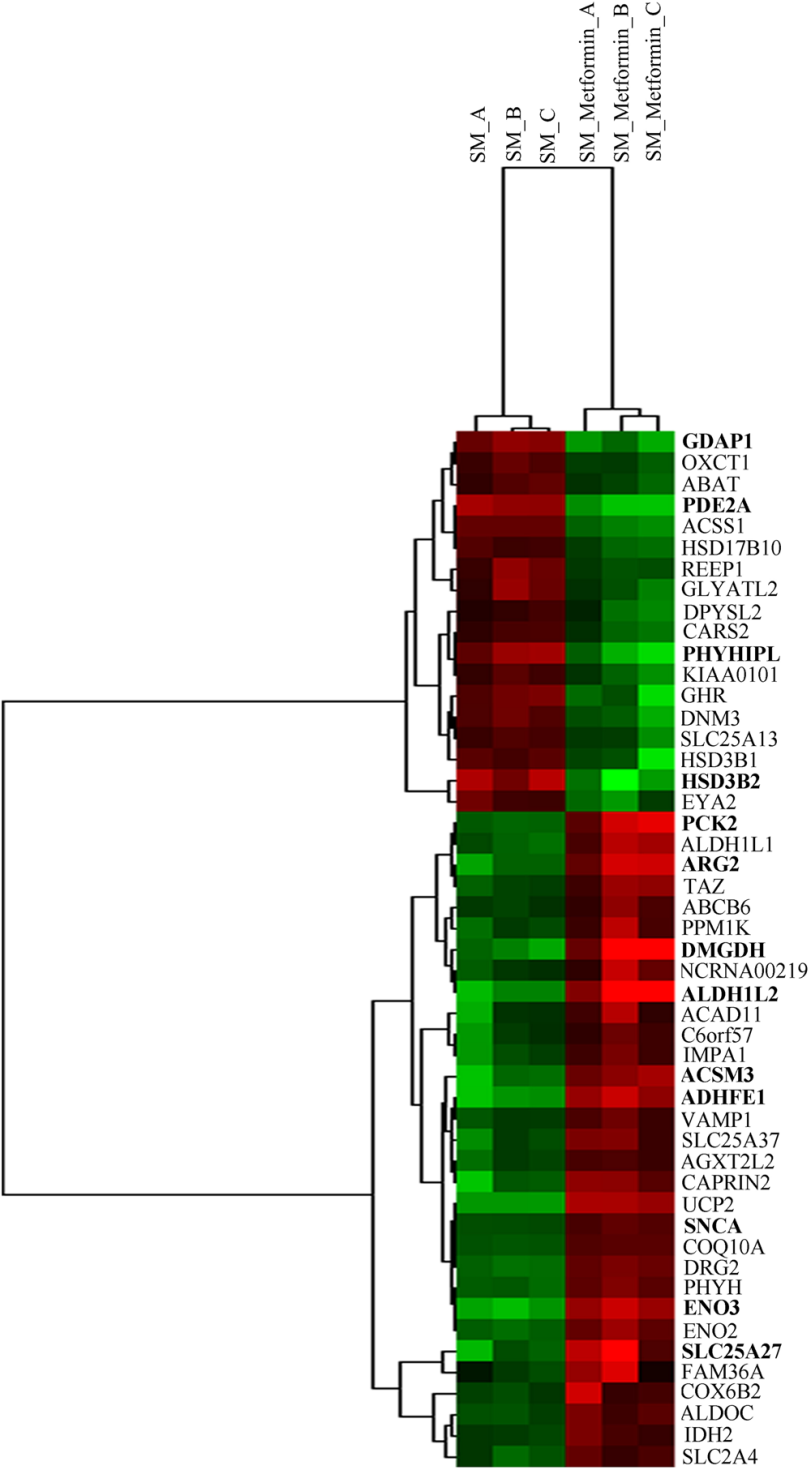
Heat map of metformin targeted genes involved in metabolic pathways of the mitochondrium. Data of mitochondrial genes were extracted from the microarray data using the GeneCodis web program. Selection was mainly based on gene product localization in the mitochondrium, few genes were added for their known involvement in glycolysis and gluconeogenesis. Differentially expressed genes (>1.5 fold) were analyzed by Cluster 3.0 and JTreeview software to generate a representative heat map. In the heat map graphic, rows show individual genes. Gene expression levels are displayed for each independent sample. Over-expression is shown in red color, under-expression in green color. Genes highlighted in bold are identified at a significance level >2.0 fold change. SM – starvation medium.

Previous studies suggested that metformin acts via mitochondrial complex I to regulate glucose homeostasis^15^ and androgen production^21^. Therefore, to study the role of mitochondria, we mapped all identified metformin-altered genes for their effect on mitochondrial metabolic pathways. First, we generated a heat map of selected differentially expressed genes (>1.5 fold, p < 0.05) that are involved in different mitochondrial metabolic processes, glycolysis and glyconeogenesis (Fig. 5). These genes were extracted from the microarray data based on their cellular localization. Selected genes were then investigated for their involvement in different metabolic pathways and mitochondrial processes using the Reactome pathway analyzer (Table 2). This analysis showed that most of the metformin regulated genes were comprised in expected metabolic processes, like the respiratory electron transport, ATP synthesis by chemiosmotic coupling and heat production by uncoupling proteins (Table 2).

**Table 2:** Reactome pathway analysis of selected differentially expressed genes under metformin treatment.

Reactome pathway analysis	Maps	SM vs Met			
		p-value	FDR	n	Genes
1	Metabolism	0.00353	0.04239	28	IDH2, ACSS1, IMPA1, **ACSM3**, **ENO3**, ENO2, ALDOC, GLYATL2, **ADHFE1**, GLUT4, COX6B2, **HSD3B2**, SLC25A37, PHYH, SLC25A13, HSD3B1, CARS2, TAZ, ACSS1, **PCK2**, UCP2, **ARG2**, ENO, HSD17B10, **SLC25A27(UCP4)**, FAM36A, OXCT1(SCOT)
2	Gluconeogenesis	0.00007	0.00665	5	**ENO3**, ENO2, **PCK2**, ALDOC, SLC25A13,
3	Glycolysis	0.00701	0.07012	3	**ENO3**, ENO2, ALDOC,
4	The fatty acid cycling model	0.00136	0.02576	2	**SLC25A27(UCP4)**, UCP2
5	Conjugation of phenylacetate with glutamine	0.0021	0.035	1	ACSM3
6	The citric acid (TCA) cycle and respiratory electron transport	0.00042	0.01393	6	IDH2, **ADHFE1**, **SLC25A27 (UCP4)**, COX6B2, UCP2, FAM36A
7	Mitochondrial Uncoupling Proteins	0.0035	0.042	2	SLC25A27, UCP2
8	Respiratory electron transport, ATP synthesis by chemiosmotic coupling, and heat production by uncoupling proteins.	0.01411	0.09967	4	**SLC25A27 (UCP4)**, COX6B2, UCP2, FAM36A

GeneGo reactome pathway analysis revealed that metformin (Met) regulated genes are involved in many different metabolic processes. The table shows the top 8 pathways and processes. The cut-off was set at >1.5 fold. Genes identified at a cut-off of >2.0 fold are given in bold. (FDR = FDR = false discovery rate; n = number of genes identified).

**Figure 6:**
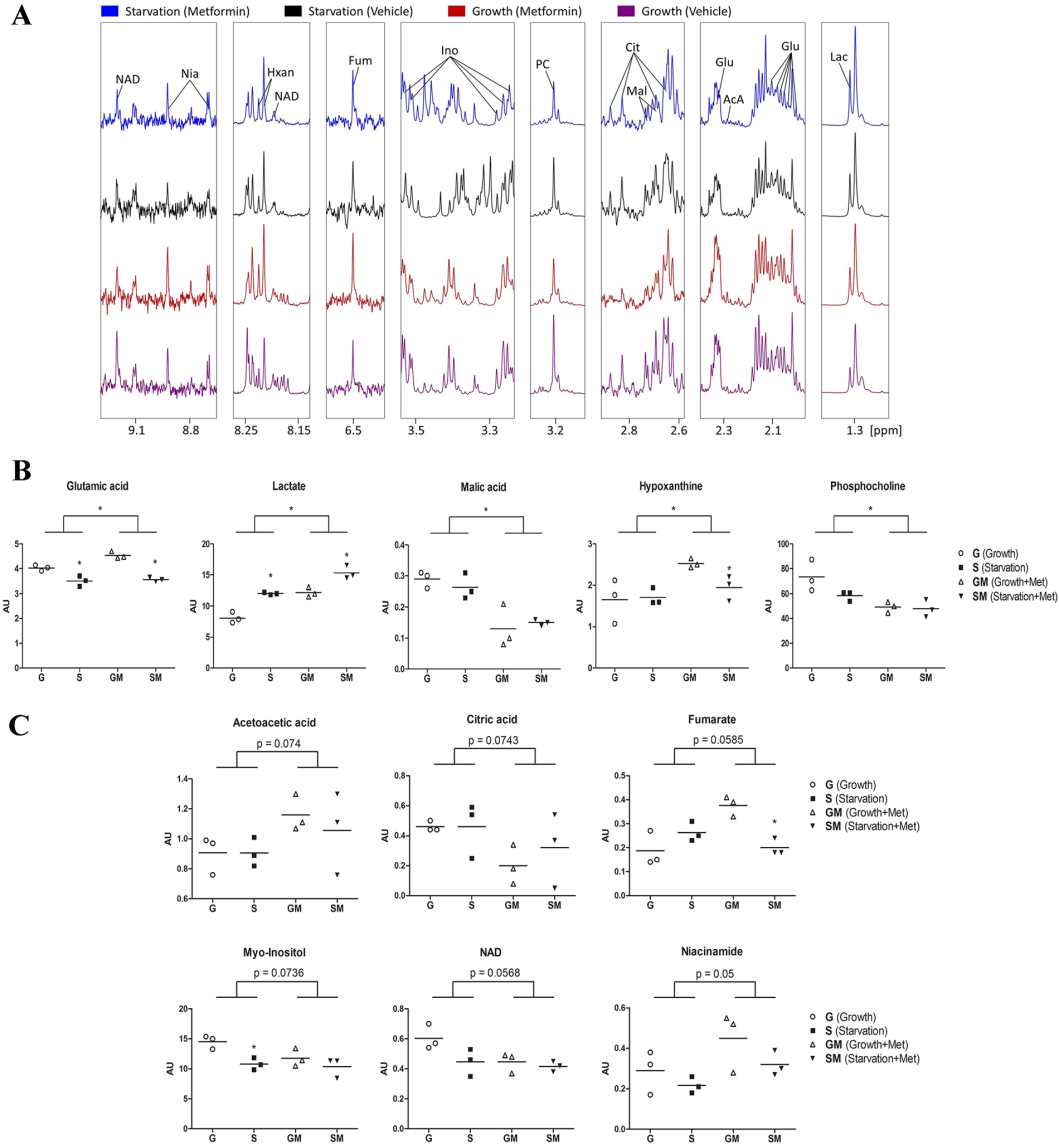
Metabolite analysis of human adrenal H295R cells using^1^H HR-MAS NMR. Cells were grown in normal (GM) or starvation medium (SM) and with or without 10 mM metformin (Met). Cell lysates were heat-inactivated and frozen before analysis by NMR spectroscopy. In total, 38 metabolites were identified (see Supplementary Table 2). (**A**) 1D spectra excerpts with assignments of selected metabolites identified in differently treated H295R cells as indicated with different colors. (**B**,**C**) Summary of metformin targeted metabolites from NMR measurements on three cell cultures for each condition showing significant (**B**) or near significant alterations (**C**). Statistical significance between groups was calculated by the two-way ANOVA test, while Student t-test was used to test significance between two items. *p < 0.05.

To confirm the metabolic changes brought by metformin treatment, we also performed NMR spectroscopy to assess the metabolome of H295R cells with and without metformin treatment directly. Thirty eight intracellular metabolites were detected in total (Supplementary Table 3). We found five intracellular metabolites significantly changed after metformin treatment. These metabolites were key substrates or intermediate compounds in basic metabolic processes such as glycolysis, gluconeogenesis and the citric acid cycle (glutamic acid p = 0.010; lactate p < 0.001; malic acid p < 0.001; hypoxanthine p = 0.018 and phosphocholine <0.005) (Fig. 6B). Another six metabolites showed near significant changes upon metformin treatment, including acetoacetic acid (p = 0.074), citric acid (p = 0.074), fumarate (p = 0.058), myo-inositol (p = 0.073), NAD (p = 0.057) and niacinamide p = 0.05) (Fig. 6C). As substrates or cofactors, these metformin altered metabolites play important roles in metabolic pathways of glycolysis, citric acid cycle, ketone metabolism and fatty acid catabolism.

### Integrated analysis of the metformin targeted metabolome and transcriptome

**Figure 7:**
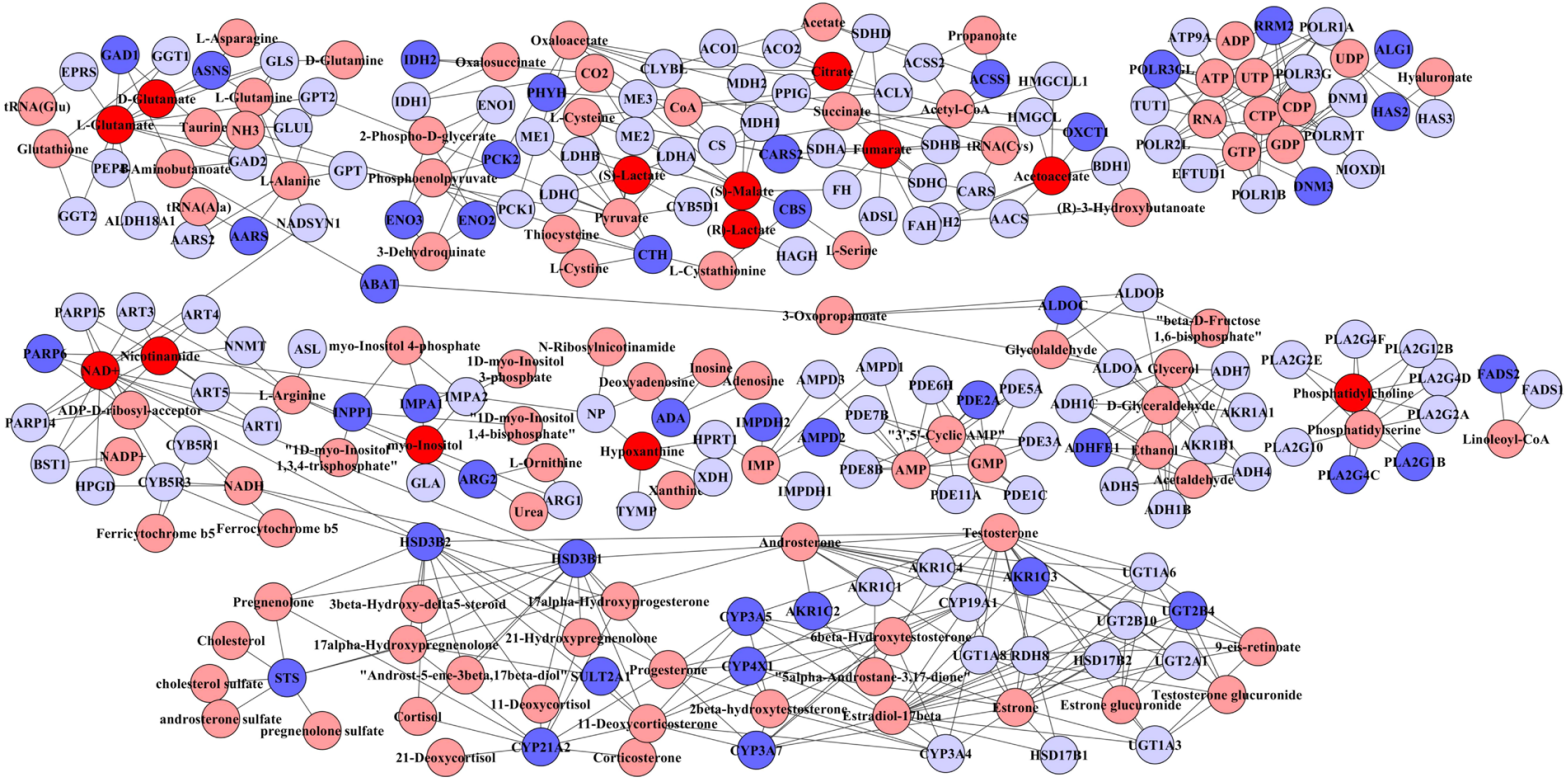
Integrative network analysis of transcriptome and metabolome data obtained from metformin treated H295R cells. The depicted network shows mitochondrial and associated metabolites and their relationship to genes changed by metformin treatment (>1.5 fold). The integrative network was generated using MetScape plugin for cytoscape. It shows the relationship between the metformin altered metabolites identified by NMR (shown in dark red) and the genes identified by microarray expression profiling of metformin treated H295R cells (dark blue). Other associated metabolites in the given network (proposed by the program) are highlighted in light red and associated genes in light blue. Interestingly, the network identified the mitochondrial metabolites NAD^+^/NADH and proposes their impact on molecules or enzymes involved in androgen biosynthesis.

Finally, we performed an integrated network analysis on the gene expression microarry data and the NMR metabolite data obtained from metformin treated H295R cells using IMPaLA. Metformin altered a wide range of cellular processes, among them mainly gene expression (regulated by epigenetic and transcription factors), signaling (e.g. WNT, serine biosynthesis), and metabolic pathways (e.g. gluconeogenesis, glucose metabolism, glycolysis, ketolysis, butanoate metabolism, lipids and lipoproteins metabolism, TCA cycle and respiratory electron transfer) (Supplementary Table 4). Steroid biosynthesis and metabolism, such as allopregnanolone biosynthesis, androgen and estrogen biosynthesis and metabolism were also on the list of processes altered by metformin. Additionally, we generated a map to visualize the integrative network for a better understanding of the relationship between the metabolites and genes targeted by metformin (Fig. 7). On this map a clear interrelationship between steroidogenesis and metabolic pathways related to energy homeostasis was visible. Interestingly, the decrease of mitochondrial NAD by metformin seems to modulate both energy/glucose metabolism and steroid/androgen biosynthesis.

## Discussion

Metformin regulates intracellular energy homeostasis and androgen metabolism in human adrenal H295R cells by a complex (signaling) network. Performing microarray gene expression and NMR spectroscopic metabolic profiling studies on metformin treated H295R cells, we were able to map the involved genes, proteins and metabolites and their broader interrelationship.

**Figure 8:**
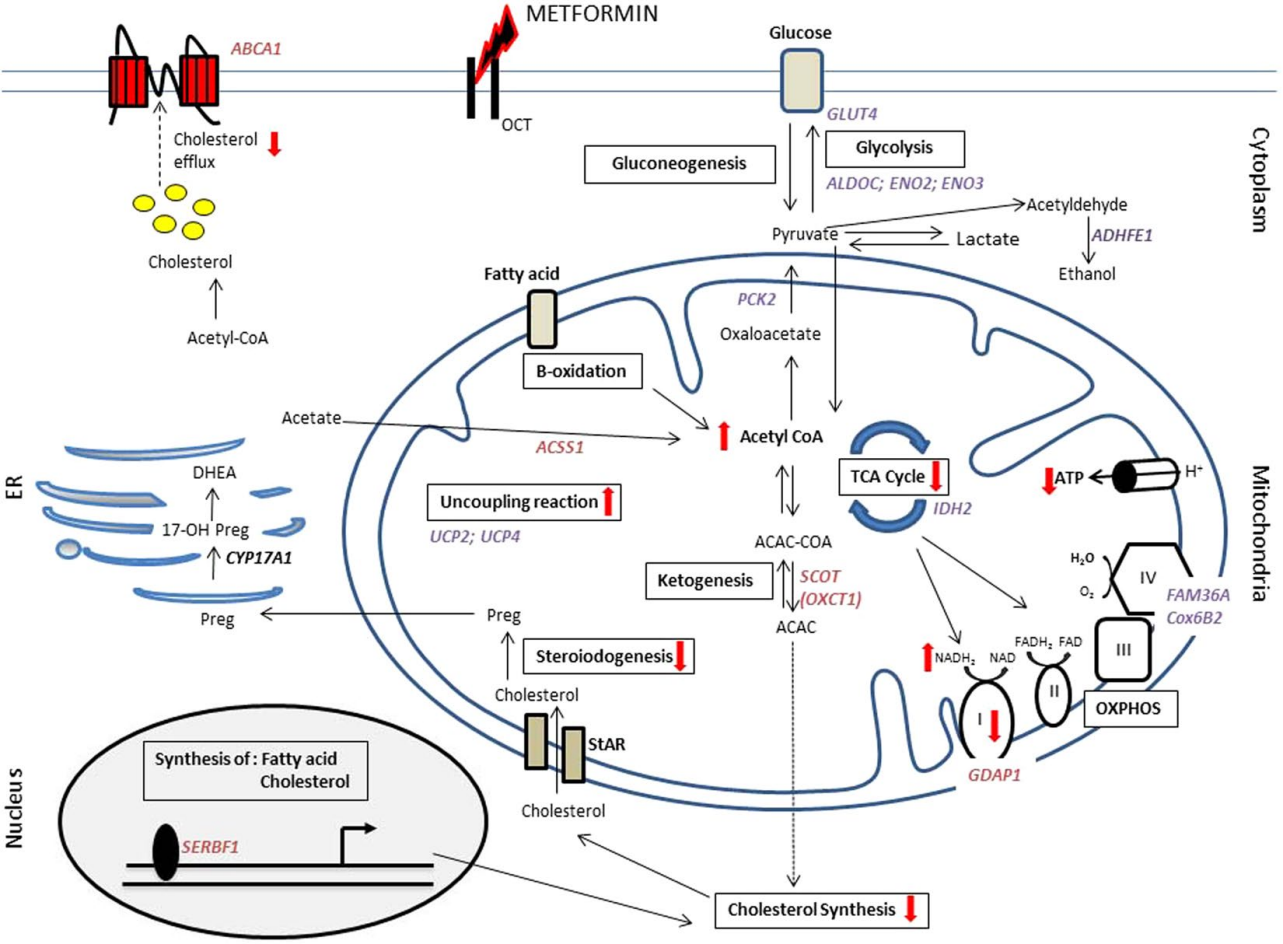
Schematic overview of effects of metformin on gene expression and metabolism in human adrenal H295R cells, focused on mitochondria and steroidogenesis. Based on the reactome pathway analysis (Table 2), we mapped metformin-altered genes into the landscape of known mitochondrial metabolic pathways. It is known that metformin is transported into cells through the OCT family of transporters. In mitochondria metformin inhibits complex I-dependent respiration and decreases ATP production. Our transcriptome data indicate that metformin increases the uncoupled respiration by upregulating UCP2 and UCP4 gene expression, and increases glycolysis by upregulating GLUT4, ALDOC, ENO2, ENO3 gene expression. This may result in an increase in lactate production and a decrease in pyruvate, confirming our metabolome data. Additionally, we found that metformin decreases SCOT gene expression, which is important for ketone body acetoacetate formation. We also found decreased expression of the SERBF1 transcription factor, which regulates fatty acid and cholesterol synthesis. This suggests a possible negative effect on cholesterol synthesis that is important for all steroidogenesis.

Most of the identified metformin modulated genes and metabolites were involved in intracellular metabolic processes such as the respiratory electron transport chain, ATP synthesis or the carbohydrate and lipid metabolism (Fig. 8). An increased expression of ENO2, ENO3 and ALDOC, which are essential for glycolysis was observed. By contrast, only one gene of gluconeogenesis, PCK2, was down-regulated by metformin. Glycolysis converts glucose to pyruvate. Pyruvate may be converted to lactate or acetaldehyde and ethanol, or may be transformed into acetyl-CoA to enter the tricarboxylic acid (TCA, Krebs) cycle. Previously an increased lactate to pyruvate ratio was found in metformin treated H295R cells^21^. The current NMR measurements confirm this finding.

We also found an increased expression of the uncoupling proteins UCP2 and UCP4 (SLC25A27) after metformin treatment. Human UCP2 is widely expressed and associated with energy expenditure and thermogenesis; it is therefore a potential therapeutic target for diabetes and obesity^33, 34^. UCP4 seems protective against oxidative stress and promotes glycolysis and ATP production in rat adrenal medulla cells^35^. Thus our data suggest that metformin increases uncoupling reactions and enhances energy expenditure.

Additionally, metformin down-regulated SERBF1, a transcriptional regulator of fatty acid and cholesterol synthesis. SREBPs activate around 30 genes directly, which are related to the synthesis and uptake of cholesterol, fatty acids, triglycerides, and phospholipids^36, 37^. Metformin also lowered OXCT1 gene expression, encoding the mitochondrial succinyl-CoA-3-oxaloacid CoA transferase (SCOT) enzyme. SCOT plays a pivotal role in extrahepatic ketone body catabolism by catalyzing the reversible transfer of coenzyme A from succinyl-CoA to acetoacetate, thereby integrating the TCA cycle, β-oxidation and de novo lipogenesis. Taken together, our data indicate that decreased expression of SERBF1 and SCOT may lead to reduced cholesterol synthesis as an effect of metformin treatment. Recently, clinical studies in patients with type 2 diabetes (T2DM) revealed that metformin treatment lowered serum LDL-cholesterol significantly^38^. Similar to our results, lower levels of phosphatitylcholines and SREBP1 were also found in metformin treated T2DM.

Our omics studies confirmed metformin’s inhibitory effect on complex I of the OXPHOS system, confirming its effect on androgen production^21^. The complex I generated NAD, which is the essential cofactor for the HSD3B2 enzyme activity, is diminished by metformin treatment and leads to lowered androgen production^39, 40^.

Metformin possesses also antineoplastic properties^41–43^. These effects of metformin have been recently reported as being mediated by cholesterol-dependent mechanisms and by altered mitochondrial metabolic processes^44^. These findings were confirmed by our enrichment analysis. But more importantly, we found that metformin altered several pivotal genes involved in cell cycle and cell proliferation. Novel cancer-related genes identified include *DPYSL3, NOV* and *IDO1*. *DPYSL3* encodes the dihydropyrimidinase-like 3 protein, which regulates cancer cell migration and adhesion *in vitro* and *in vivo* ^45^, and may facilitate malignant behavior^46^. The *NOV* (nephroblastoma overexpressed) gene (also known as *CCN3*) is a new member of the CCN family of secreted, extracellular matrix (ECM)-associated regulatory factors that are involved in internal and external cell signaling and play a role in angiogenesis and osteogenesis, and in the control of cell proliferation and differentiation^47, 48^, even against human adrenocortical tumor cells^49^. Finally, the *IDO1* gene encodes the indoleamine 2,3-dioxygenase enzyme, which catalyzes the initial and rate limiting step of the tryptophan catabolism, which plays an important role in the regulation of the immune response and in tumoral immune resistance^50^. Thus metformin may not only possess anti-cancer properties, but may also have positive immunmodulatory effects.

However, recent transciptome studies on metformin’s effects in pancreatic cancer and prostate cancer cells revealed different, non-overlapping gene expression profiles compared to our study, indicating that metformin exerts its effects in a cell-/tissue-specific manner^51, 52^. By contrast, the gene expression profile of H295R cells after treatment with the adrenolytic agent mitotane showed similar changes as with metformin, namely alterations in the expression of ALD1L2, GDF15, TRIB3, HSD3B1, HSD3B2 and CYP21A1^53^.

With respect to antineoplastic metabolic effects in breast cancer cells, metformin was also reported to possess characteristics of an antifolate chemotherapeutic drug^54^. Metformin treatment resulted in reduced metabolism of tetrahydrofolate carrying essential one-carbon units for *de novo* purine and pyrimidine synthesis and led to a depletion of glutathione. But in H295R cells, metformin had no effect on glutathione.

Our network analysis also showed connections of metformin to PCOS related genes and metabolites. For example, under metformin treatment expression of the membrane protein CYR61 was enhanced. CYR61 is an estrogenic biomarker of the human endometrium and as such an early signal for the development of endometrium hyperplasia or adenocarcinoma in PCOS women^55^. In our studies, we found an interconnection between CYR61, the alpha/beta integrin signaling cascades, the activated c-Src kinase and the androgen receptor upon metformin treatment^56, 57^. By contrast, metformin down-regulated versican (VCAN), an extracellular proteoglycan protein. This protein, which is expressed in periovulatory granulosa cells and located within the expanding matrix of the cumulus oocyte complex, seems to play a role in ovulatory dysfunction and PCOS^58^. By contrast, we did not find significant alterations in risk genes recently identified in a large genome-wide association study (GWAS) performed in PCOS women in the Han Chinese population^59^.

Metformin also affected GPCRs and depending protein kinase signaling cascades. Specifically, expression of MC2R and GnRHR were altered. The hypothalamic gonadotropin-releasing hormone (GnRH) is a central regulator of the mammalian reproductive system. It binds to its specific GnRH receptor (GnRHR) in the pituitary gonadotrophs and triggers the synthesis of luteinizing hormone (LH) and follicle-stimulating hormone (FSH). Secreted LH and FSH bind to their cognate receptors in the gonads to control reproductive functions including the production of sex steroids^60^. On the other hand, the melanocortin 2 receptor (MC2R) is specifically expressed in the human adrenal cortex and selectively stimulated by ACTH to regulate adrenal glucocorticoid and androgen production^61, 62^.

Finally, our studies identified novel targets of metformin involved in sex steroid production and regulation, specifically the genes for HSD17B14, AKR1C3 and SULT2A1. HSD17B14 (17β-hydroxysteroid dehydrogenase 14) is expressed in various human tissues; it converts estradiol and testosterone into the less active steroid metabolites estrone and androstenedione, respectively^63^. AKR1C3 (aldoketoreductase type 1C3, also known as HSD17B3) plays a major role in androgen production of the classic and the recently described alternative, backdoor pathway. It plays a key role in prostate cancer and other endocrine disorders^64^. The cytosolic SULT2A1 (sulfotransferase 2A1) catalysis the sulfation of dehydroepiandrosterone (DHEA) to DHEA sulfate (DHEA-S) in the human liver and adrenals, but it is also responsible for the sulfonation of other hydroxysteroids such as pregnenolone, cholesterol and bile acids^65, 66^. In addition, metformin was also found to regulate KCND2 gene expression. This is an interesting finding as KCND2 encodes a voltage-dependent K+ channel, which mediates a rapidly inactivating outward potassium current that regulates diverse cell functions. This ion channel has been previously identified to be steroid-sensitive in human ovaries, providing evidence for non-genomic steroid actions^67^. Our data now suggest that this channel may be modulated by metformin.

Limitations of our study are manifold. We used a cell model, which is a simplistic system compared to a whole human being. In addition, the H295R cell line is an immortalized cancer cell line. However, this cell line has been used to explore human steroidogenesis for decades with great success^27, 68^. It is therefore well characterized. In our model setting we compared different cell conditions after specific manipulations (namely starvation and metformin treatment) looking for changes hinting underlying regulatory mechanisms. We are convinced that our model produced valid results, because in line with our findings, we found many confirmatory data in the literature from both *in vitro* and *in vivo* studies as discussed. For metabolomics analysis we used HR-MAS-NMR and determined 38 intracellular metabolites on a relatively small number of samples. The statistical evaluation to determine differences of single metabolites between groups was therefore limited (except for lactate); rather only differences of metabolic pathways/processes could be assessed. Another limitation was that we did not separate the amount of metabolite in cell compartments, such as ER, mitochondria and cytosolic compartment. We measured the total intracellular content. Future experiments could direct towards using fluorescence-labeled metabolites and cofactors to assess sub-cellular contents *in situ* with live measurement.

In summary, omics studies of metformin treated adrenal H295R cells allowed to identify the complex cellular network of metformin’s action, specifically between the energy homeostasis, steroidogenesis and the reproductive system.

## Materials and Methods

### Cell cultures and treatments

Human adrenocortical NCI-H295R cells were obtained from American Type Culture Collection (ATCC; CRL-2128). H295R cells were cultured in DMEM/Ham’s F-12 medium containing L-glutamine and 15 mM HEPES medium (GIBCO, Paisley, UK) supplemented with 5% NU-I serum (BD Biosciences, Allschwil, Switzerland), 0.1% insulin, transferrin, and selenium (100 U/ml; GIBCO), penicillin (100 U/ml; GIBCO) and streptomycin (100 µg/ml; GIBCO). The serum-free starvation medium consisted of DMEM/Ham’s F-12 medium, penicillin and streptomycin (100 µg/ml; GIBCO). For microarray experiments and for steroid profiling experiments, cells were first grown in normal growth medium for 24 h. Medium was then replaced, and cells were grown in the presence of 10 mM metformin in serum-free medium for 48 h^21^.

### Steroid profiling

Steroid profiling was performed after growing cells in 6 well plates and adding labelled 100,000 cpm [^3^H] pregnenolone to the culture medium for 90 min. Steroids were then extracted from cell supernatants and separated by thin layer chromatography (TLC) as previously described^21, 23^. Steroid conversion of pregnenolone into metabolites was calculated as percentage of total radioactivity incorporated into specific products and is therefore given as %. Specific products were identified by running standards in parallel.

### Microarray analysis for gene expression profiling

Total RNA was isolated and purified with the RNeasy Micro kit (Qiagen GmbH, Hilden, Germany) from cultured H295R cells grown under normal growth, serum starvation and metformin conditions. Microarray experiments were carried out on three independent experiments with the GeneChip Human Gene 1.0 ST arrays (Affymetrix Inc., Santa Clara, CA, USA) at the Genomic Technologies Facility (GTF) of the University of Lausanne, Switzerland. Data are available in the repository of GEO profiles at NCBI. Data were analyzed using the Affymetrix Power Tools package (for 1.0-ST arrays). All statistical analyses were performed using the free high-level interpreted statistical language R and various Bioconductor packages (http://www.Bioconductor.org). For analysis, the *p*-values were adjusted for multiple testing with the Benjamini and Hochberg’s method to control for false discovery rate (FDR). Probe sets showing at least a ±1.5-fold change and a FDR < 0.05 were considered significant. Hierarchical clustering was carried out using the Cluster 3.0 and Jtreeview software for creating representative heat maps.

### Enrichment and functional data analysis

Transcripts with an adjusted *p*-value < 0.05 and a fold change >1.5 were considered differentially expressed. Differentially expressed transcripts were further analyzed using the GeneGo MetaCore analysis software (GeneGo Inc., Minneapolis, MN, USA) to identify their involvement in specific biological processes, pathways and process networks as well as their involvement as diseases biomarkers. MetaCore contains interaction networks, which are based on manually annotated and regularly updated databases. We used the auto-expand algorithm to identify connected gene networks in a set of differentially expressed genes of interest from our experimental data. The auto-expand algorithm begins with a number of root nodes, which is set by the user, and builds subnetworks across the uploaded data sets and adjoining neighbors. The expansion stops when the subnetworks meet. Each connection identifies a direct, experimentally confirmed, physical interaction between genes. The auto-expand algorithm allows looking up one or more genes of interest and identifies regulatory cascades that lead to or from (a) gene(s) of interest. The networks can be visualized graphically as nodes (genes or proteins) and edges (the relationship between genes or proteins). Additionally, we used the web program GeneCodis (http://genecodis.dacya.ucm.es) and the Reactome Pathway database (http://www.reactome.org) to identify further information on genes involved in biological processes and pathways.

### Quantitative real time PCR (qRT-PCR)

Total RNA was extracted from adherent H295R cells cultured under normal growth, serum starvation and metformin treatment conditions using the TRIzol reagent according to the manufacturer’s instructions (Invitrogen Life Technologies, Carlsbad, CA, USA), followed by reverse transcription using the Improm RNA transcriptase kit (Promega, Madison, WI, USA) as previously described^23, 26^. Quantitative RT-PCR analysis was performed on the 7500-Fast real-time PCR System (Applied Biosystems, Foster City, CA, USA) using ABsolute SYBR Green Mix (ABgene; Thermo Fisher scientific, Waltham, MA, USA). As endogenous control, the GAPDH gene was used. Fold change in gene expression was calculated by the 2^−ΔΔCt^ method^69^.

### Sample preparation and NMR measurements

For metabolomics analysis, H295R cells were cultured under normal growth and starvation conditions with and without 10 mM metformin treatment. After 48 h of incubation, cells were washed with PBS, trypsinized and collected. Consequently, each sample was further washed three times with 1 mL PBS and cells were counted using a Trypan blue exclusion assay in a standard hemocytometer, showing a cell viability of over 90%. Ten million cells were prepared in 55 μL D_2_O-based 10 mM PBS (pH 7.4). The cell suspension was then sonicated for 30 seconds and dipped into liquid nitrogen (15 sec) for shock freezing; this procedure was repeated three times. Finally, the cell metabolism was inactivated by heating the sample at 70 °C for 20 min. The cell suspension was then transferred into a standard zirconium magic angle spinning (MAS) rotor using a 50 μL insert.

NMR measurements were carried out on three independent cell cultures for each condition. ^1^H High Resolution Magic Angle Spinning (HR-MAS) NMR experiments were performed on a Bruker Avance II spectrometer (Bruker Karlsruhe, Germany) operating at a resonance frequency of 500.13 MHz using a 4 mm HR-MAS dual inverse^1^H/^13^C probe equipped with a deuterium lock channel and with a magic angle gradient. Bruker TOPSPIN software (version 3.2, patch level 5) was used to acquire and process the NMR data. The measurements were performed at a nominal temperature of 276 Kelvin and at a magic angle spinning speed of 3 kHz. The PROJECT sequence^70^ was used with presaturation of the water signal. The measurements were performed at an echo time (TE) of 400 ms (300 loops, rotor-synchronized interpulse delay of 0.33 ms) and a recycle delay of 4 s. Each PROJECT spectrum was acquired applying 1024 transients, a spectral width of 12 ppm and a data size of 32 K points. For all spectra, the co-added free induction decays (FIDs) were exponentially weighted with a line broadening factor of 1.0 Hz, Fourier-transformed, phased and frequency calibrated to the left peak of the lactate doublet (1.324 ppm) to obtain the^1^H NMR spectra. Spectral assignment was based on literature^71^, data obtained from the Human Metabolome Database (HMDB)^72^ and our own additional 2D correlation spectroscopy (^1^H^1^H-TOCSY) measurements. A total of 171 buckets (between 0.9 and 9.2 ppm) were selected, with a variable size according to the peak width. Spectral regions comprising only noise as well as spectral regions with contributions from metformin were excluded from all analyses. The buckets were normalized by probabilistic quotient normalization (PQN)^73^.

### Integrated analysis of transcriptome and metabolome data

We performed integrated molecular pathway analysis of transcriptomics and metabolomics data using the IMPaLA (http://impala.molgen.mpg.de/) web tool^74, 75^. This tool performs over-representation or enrichment analysis with user-specified lists of metabolites and genes using over 3000 pre-annotated pathways from 11 databases. Here we performed an over-representation analysis based on our list of 11 metabolites and 693 differentially expressed genes altered by metformin treatment. In addition, visualization of networks and interpretation of transcriptomics and metabolomics data were performed by using MetScape 3 (http://metscape.ncibi.org/), a plugin for Cytoscape (http://www.cytoscape.org/)^76–78^. The tool MetScape allows users to analyze experimental data from gene and/or metabolite lists in order to integrate and visualize common networks in the context of the human metabolism.

### Statistical analyses

Statistical analyses were performed with Microsoft Excel and Prism 6 (Graph Pad Software, Inc. San Diego, CA, USA). For data analyses of steroid profiling and qRT-PCR, statistical differences between values were calculated using the Student’s t-test. For analysis of the metabolome, statistical analyses were carried out by the two-way (factorial) ANOVA test, with metformin treatment and medium used as two independent variables. P-values below 0.05 were considered statistically significant. To calculate for the effect of starvation, analysis between two data sets was performed by the Student’s t-test. Quantitative data represent the mean of at least three independent experiments, error bars indicate the mean ± SD. Significance was set at *p < 0.05 and **p < 0.01.

## Electronic supplementary material

Supplementary Information in Text, Table and Figure
