# Supplementary material for: Combined transcriptome and metabolome analyses of metformin effects reveal novel links between metabolic networks in steroidogenic systems
Source: Sci Rep. 2017 Aug 17;7:8652. doi: 10.1038/s41598-017-09189-y (PMC5561186; doi:10.1038/s41598-017-09189-y)
Supplement: Supplementary file 1 — Supplementary Information in Text, Table and Figure [file 41598_2017_9189_MOESM1_ESM.pdf]

Supplementary Information to:

Combined transcriptome and metabolome analyses of metformin effects reveal novel links between metabolic networks in steroidogenic systems

Sameer S. Udhane, Balazs Legeza, Nesa Marti, Damian Hertig, Gaëlle Diserens, Jean-Marc Nuoffer, Peter Vermathen, Christa E. Flück

List of items:

Supplementary Figure S1 - Effect of starvation, growth conditions and metformin treatment on steroidogenesis in human adrenal H295R cells.

Supplementary Table S1: List of genes significantly altered by metformin (Met) treatment.

Supplementary Table S2: Enrichment analysis of differentially expressed genes involved in diseases.

Supplementary Table S3: Intracellular metabolites detected by nuclear magnetic resonance spectroscopy (NMR) in human adrenal H295R cells under metformin treatment.

Supplementary Table S4: Integrated pathway analysis of transcriptome and metabolomics data for H295R cells treated with metformin.

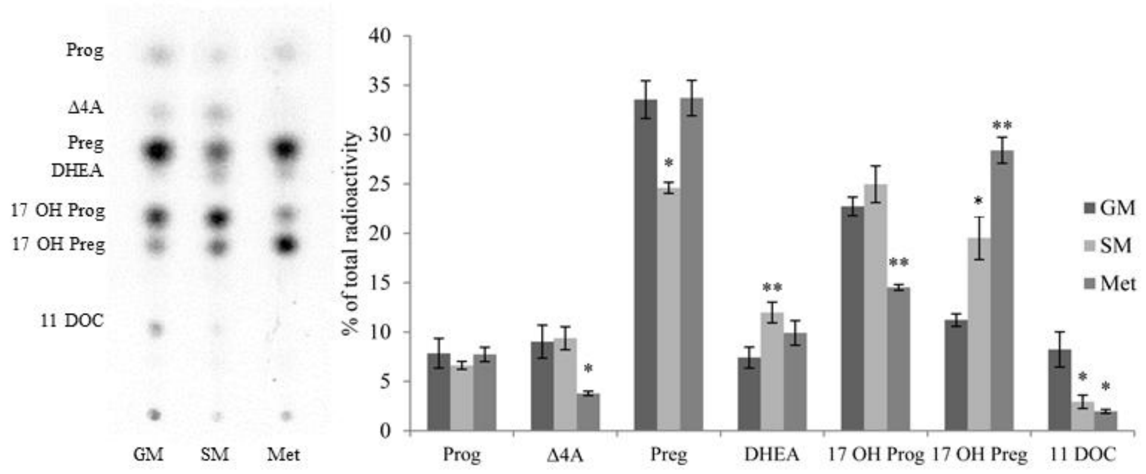

Supplementary Figure S1. Effect of starvation, growth conditions and metformin treatment on steroidogenesis in human adrenal H295R cells. Cells were grown for 48 h under starvation conditions (SM) without and with treatment of metformin (Met). Steroid production was labeled with [3H] pregnenolone for 90 min. Extracted steroids were resolved by thin layer chromatography (TLC). Steroid profiles were compared to normal growth conditions (GM). A. Representative TLC showing the steroid profile from H295R cells grown under the three conditions. B. Quantitative analysis. Data are the mean ± SD of three independent experiments. * $p < 0.05$, ** $p < 0.01$. Prog, progesterone; Δ4A, androstenedione; Preg, pregnenolone; DHEA, dehydroepiandrostendione; 17OH Prog, 17α-hydroxyprogesterone; 17OH Preg, 17α-hydroxypregnenolone; 11 DOC, 11-deoxycortisol.

Supplementary Table S1: **List of genes significantly altered by metformin (Met) treatment**. 104 genes were identified by microarray analysis with a fold change cut-off at 2.0 and an adjusted p-value < 0.05.

	Gene Accession	Gene. Symbol	Fold Change	Gene.Description
1	NM_000345	SNCA	2,3713	synuclein, alpha (non A4 component of amyloid precursor)
2	NM_006855	KDELR3	2,2077	KDEL (Lys-Asp-Glu-Leu) endoplasmic reticulum protein retention receptor 3
3	NM_001448	GPC4	3,3950	glypican 4
4	NM_002599	PDE2A	-2,4528	phosphodiesterase 2A, cGMP-stimulated
5	NM_003513	HIST1H2AB	-2,6682	histone cluster 1, H2ab
6	NM_001935	DPP4	3,0335	dipeptidyl-peptidase 4
7	NM_207299	LPPR1	-2,9661	lipid phosphate phosphatase-related protein type 1
8	NM_001102559	PPAPDC1B	2,1017	phosphatidic acid phosphatase type 2 domain containing 1B
9	NM_005822	RCAN2	-2,0400	regulator of calcineurin 2
10	NM_016546	C1RL	2,0221	complement component 1, r subcomponent-like
11	NM_003544	HIST1H4B	-2,2526	histone cluster 1, H4b
12	NM_004644	AP3B2	-2,1846	adaptor-related protein complex 3, beta 2 subunit
13	AK096213	FLJ38894	-2,2366	hypothetical protein LOC646029
14	NM_152666	PLD5	-3,6922	phospholipase D family, member 5
15	NM_004388	CTBS	2,2668	chitobiase, di-N-acetyl-
16	NM_001048201	UHRF1	-2,4070	ubiquitin-like with PHD and ringfinger domains 1
17	NM_018972	GDAP1	-2,1010	ganglioside-induced differentiation-associated protein 1
18	NM_021062	HIST1H2BB	-2,5979	histone cluster 1, H2bb
19	NM_015187	SEL1L3	2,8504	sel-1 suppressor of lin-12-like 3 (C. elegans)
20	NM_006216	SERPINE2	4,3213	serpin peptidase inhibitor, clade E (nexin, plasminogen activator inhibitor type 1), member 2
21	NM_152996	ST6GALNA C3	-2,3839	ST6 (alpha-N-acetyl-neuraminyl-2,3-beta-galactosyl-1,3)-N-acetylgalactosaminide alpha-2,6-sialyltransferase 3
22	NM_001946	DUSP6	-3,0078	dual specificity phosphatase 6
23	NM_001976	ENO3	2,4933	enolase 3 (beta, muscle)
24	NM_000500	CYP21A2	-2,3618	cytochrome P450, family 21, subfamily A, polypeptide 2
25	AK095259	LOC439938	3,5324	hypothetical LOC439938
26	NM_003529	HIST1H3A	-2,0910	histone cluster 1, H3a
27	NM_005824	LRRC17	-2,2439	leucine rich repeat containing 17
28	NM_001029858	SLC35F1	-2,2465	solute carrier family 35, member F1
29	NM_002508	NID1	2,005	nidogen 1
30	NM_005622	ACSM3	2,1130	acyl-CoA synthetase medium-chain family member 3
31	NM_017770	ELOVL2	-2,1651	elongation of very long chain fatty acids (FEN1/Elo2, SUR4/Elo3, yeast)-like 2
32	NM_024677	NSUN7	2,0845	NOP2/Sun domain family, member 7
33	NM_003535	HIST1H3J	-2,2111	histone cluster 1, H3j
34	NM_003521	HIST1H2BM	-2,0530	histone cluster 1, H2bm
35	NM_001008540	CXCR4	-2,4110	chemokine (C-X-C motif) receptor 4

36	NM_001012758	NUDT17	2,3340	nudix-type motif 17
37	NM_004385	VCAN	-3,2544	versican
38	NM_012242	DKK1	-2,4866	dickkopf homolog 1 (Xenopus laevis)
39	NM_001042483	NUPR1	3,2682	nuclear protein, transcriptional regulator, 1
40	NM_016246	HSD17B14	2,2892	hydroxysteroid (17-beta) dehydrogenase 14
41	NM_000351	STS	-2,2465	steroid sulfatase (microsomal), isozyme S
42	NM_021190	PTBP2	-2,1106	polypyrimidine tract binding protein 2
43	NM_144650	ADHFE1	2,4320	alcohol dehydrogenase, iron containing, 1
44	NM_015515	KRT23	2,1909	keratin 23 (histone deacetylase inducible)
45	NM_032439	PHYHIPL	-2,2636	phytanoyl-CoA 2-hydroxylase interacting protein-like
46	NM_024593	EFCAB1	2,2394	EF-hand calcium binding domain 1
47	NM_001165931	RRM2	-2,1431	ribonucleotide reductase M2
48	NR_024101	FLJ35776	2,0741	hypothetical LOC649446
49	NM_032717	AGPAT9	2,5098	1-acylglycerol-3-phosphate O-acyltransferase 9
50	NM_017826	SOHLH2	2,0105	spermatogenesis and oogenesis specific basic helix-loop-helix 2
51	NM_001172	ARG2	2,2219	arginase, type II
52	NM_058173	MUCL1	-2,0578	mucin-like 1
53	NM_017938	FAM70A	-2,2253	family with sequence similarity 70, member A
54	NM_003716	CADPS	-2,6442	Ca++-dependent secretion activator
55	NM_006790	MYOT	2,3394	myotilin
56	NM_000198	HSD3B2	-2,5164	hydroxy-delta-5-steroid dehydrogenase, 3 beta- and steroid delta-isomerase 2
57	NM_181671	PITPNC1	2,3968	phosphatidylinositol transferprotein cytoplasmic 1
58	NM_000406	GNRHR	-3,2313	gonadotropin-releasing hormone receptor
59	NM_003627	SLC43A1	2,3216	solute carrier family 43, member 1
60	NR_006880	SNORD3A	-2,4048	small nucleolar RNA, C/D box 3A
61	NM_004563	PCK2	2,1211	phosphoenolpyruvate carboxykinase 2 (mitochondrial)
62	NM_001963	EGF	2,0653	epidermal growth factor
63	NM_198947	FAM111B	-2,5804	family with sequence similarity 111, member B
64	NM_057749	CCNE2	-2,2140	cyclin E2
65	NM_015236	LPHN3	-2,0613	latrophilin 3
66	NM_173521	C9orf84	-2,0803	chromosome 9 open reading frame 84
67	NM_032566	SPINK7	2,6547	serine peptidase inhibitor, Kazal type 7 (putative)
68	NM_024579	C1orf54	2,5923	chromosome 1 open reading frame 54
69	NM_004748	CCPG1	2,1331	cell cycle progression 1
70	NM_152310	ELOVL3	-2,6618	elongation of very long chain fatty acids (FEN1/Elo2, SUR4/Elo3, yeast)-like 3
71	NM_003525	HIST1H2BI	-2,0189	histone cluster 1, H2bi
72	BX648100	C21orf15	-2,0770	chromosome 21 open reading frame 15
73	NM_000369	TSHR	2,0564	thyroid stimulating hormone receptor
74	NM_213655	WNK1	2,1726	WNK lysine deficient protein kinase 1
75	NM_001004439	ITGA11	2,3499	integrin, alpha 11
76	NM_080596	HIST1H2AH	-2,0339	histone cluster 1, H2ah
77	NM_014157	CCDC113	2,0015	coiled-coil domain containing 113
78	NM_001034173	ALDH1L2	3,4838	aldehyde dehydrogenase 1 family, member L2
79	NM_153234	LIX1	-2,2262	Lix1 homolog (chicken)

80	NM_001013732	C6orf138	-2,1391	chromosome 6 open reading frame 138
81	NM_000529	MC2R	2,0209	melanocortin 2 receptor (adrenocorticotropic hormone)
82	NM_001099	ACPP	-2,1171	acid phosphatase, prostate
83	NM_021158	TRIB3	3,1307	tribbles homolog 3 (Drosophila)
84	NR_026835	FLJ37201	2,0655	tigger transposable element derived 2 pseudogene
85	NM_001195053	DDIT3	2,8371	DNA-damage-inducible transcript 3
86	NM_032765	TRIM52	2,0640	tripartite motif-containing 52
87	NM_013391	DMGDH	2,9974	dimethylglycine dehydrogenase
88	NM_001033719	ZNF404	2,3264	zinc finger protein 404
89	NM_144975	SLFN5	2,1292	schlafen family member 5
90	NR_002722	ZNF204P	2,0929	zinc finger protein 204, pseudogene
91	BC008975	PCID2	2,7073	PCI domain containing 2
92	NM_003090	SNRPA1	2,0964	small nuclear ribonucleoprotein polypeptide A'
93	NM_017697	ESRP1	2,3390	epithelial splicing regulatory protein 1
94	NM_004864	GDF15	3,2444	growth differentiation factor 15
95	NR_033752	LOC344887	2,4298	NmrA-like family domain containing 1 pseudogene
96	NM_001162383	ARHGEF2	2,1991	Rho/Rac guanine nucleotide exchange factor (GEF) 2
97	NM_052966	FAM129A	2,3777	family with sequence similarity 129, member A
98	NM_004277	SLC25A27	2,3873	solute carrier family 25, member 27
99	NM_002133	HMOX1	2,2203	heme oxygenase (decycling) 1
100	AF001542	MALAT1	2,0732	metastasis associated lung adenocarcinoma transcript 1 (non-protein coding)
101	NR_003043	SNORD49B	2,0059	small nucleolar RNA, C/D box 49B
102	NR_002755	RNU5D	-2,1293	RNA, U5D small nuclear
103	NR_029506	MIR32	2,1706	microRNA 32
104	BC028919	TOB2	-2,0291	transducer of ERBB2, 2

Supplementary Table S2: **Enrichment analysis of differentially expressed genes involved in diseases.** Differentially expressed genes by metformin (Met) treatment were analyzed by the GeneGo Metacore software tool setting the fold change level at >1.5 (>2.0 fold in bold). Top 10 groups of diseases are listed. (FDR= false discovery rate; n= number of genes identified)

Enrichement by Diseases (by biomarkers)		SM vs Met			
	Diseases	p-value	FDR	n	Genes
1	Breast Diseases	4.357E-16	3.431E-13	337	SYP, FAM120C, CRMP2, RHCG, PLA2, cPLA2, C5orf4, Stromalins 1/2, STAG1, DKK2, Histone H1, ENO, **ENO3**, HIST1H3D, Histone H3.1, DRIM, CD86, UAP1L1, HIST1H2AE, ALG1, **Glypican-4**, c-Myc, DTL (hCdt2), SMP30, Neuropilin-1, YPEL2, ULBP2, C9orf72, TMEM45A, CARS2, GPNMB (Osteoactivin), CD101, SIA7C, UHRF1BP1, UBE1L, C14orf174, CBS, MIF4GD, Dynamin, SSAT, Rap1GAP1, **PPCKM**, SGK3, CDC45L, Thrombospondin 3, CDC25A, CDC25, FADS2, NELL2, PIAS3, C/EBP zeta, **FUS/DDIT3 fusion protein**, C/EBP, DAX1, CCL2, FTL, DGCR14, GRAF, PDF, DAGK, DGKH, PLEKHG1, **PLD5**, Galpha(s)-specific hormone protein GPCRs, **TSH receptor**, XEDAR, UCHL1, MCM2, **ADHFE1**, PPAPDC1B, ADA, Galpha(i)-specific amine GPCRs, NAV1, **ELOVL2**, HSD3B1, LANCL2, DIAC, R3HDM2, Galpha(i)-specific peptide GPCRs, Galpha(q)-specific peptide GPCRs, NPY1R, NR3A, WDR76, R-Ras, KCNH8, ARS2, CARD6, Tubulin gamma, Tubulin gamma 2, FTDH, NTN4, Ki-1/57, p21, C/EBPbeta, Cadherin 12, **DPP4**, LIFR, Plastin, USP45, Stathmin 3, HIST1H2AK, Histone H2, HIST1H2AJ, Histone H2A, DALRD3, Histone H4, AKR1C2, WISP-3, **ITGA11**, XBP1, CDC14b, Histone H2B, HIST1H2BN, Substance P, Substance P extracellular region, Neurokinin A, Neuropeptide K, Neuropeptide gamma, Na(v) I alpha, Fibrillin 2, Fibrillin, **Cyclin E2**, HAS, HAS2, DEC1 (Stra13), **SLC35F1**, HIST2H2AC, CCDC141, AGTR1, FUSIP1, ZNF608, EYA2, SLC22A17, DUS4L, SLC9A2, CLIP170, AOF1, AHR, PTCHD4, CCDC104, HIST1H2BG, HIST1H2AD, RBM3, MaxiK alpha subunit, SRX1, SCOT, **SLC43A1**, SLC25A13, U5-102 kDa, SH3PXD2B, GLUT4, MOSPD2, MAML2, CRMP4, IL23R, CACNA1 L-type, ALDOC, PDE, **PDE2A**, ZNF577, AARS, Galpha(s)-specific peptide GPCRs, ENPP1, Annexin I, TLE, **ALDH1L2**, RNF213, TGF-beta 2, TGF-beta, Tafazzin, MCM7, YY2, Casein kinase I, MMP-24, MRPS7, RBM14, ZNF382, Angiomotin (AMOT), Cadherin 11, Galpha(i)-specific metabotropic glutamate GPCRs, mGluR7, SLC19A2, CYP4X1, **UHRF1**, C1QTNF2, Deoxyribonuclease II, TMEM74, Dynein 2, cytoplasmic, light intermediate chain, nAChR alpha, nAChR alpha-5, **DKK1**, UTP14A, PHF7, JPO1, **SLFN5**, GRO-2, PPAP, Small RR subunit, **RRM2**, CYP3A7, RIT, Phosphatase regulator (inhibitor), SREBP1 precursor, SREBP1 (Golgi membrane), SREBP1 (nuclear), B4GT4, Heme oxygenase 1, FLJ21986, AP-3

					beta subunits, **AP3B2**, IMD2, GHR, BAF57, SPARCL1, CYP3A5, **LRRC17**, ENO2, Caspr1, PSMC3, **MUCL1**, PKC, PKC-eta, CACNA1H, CACNA1 T-type, CACNG, Stargazin, DGCR8, GPR155, RASGRF1, AGXT2L2, CTH, **Versican**, Versican proteoglycan, STAG3L3, **ELOVL3**, HZwint-1, TEX15, TBL1X, FSD1, SLC17A7, **ARHGEF2**, PGM2L1, **EGF**, Exportin-T, DAF, Galpha(i)-specific cannabis GPSRs, USP52, Lingo1, P4HA2, P4HA, PARC, COL15A1, PARP-6, LAMG1, HEI10, Carmil, M2GD, ABCA1, Claspin, NHE7, MR1, p56 KKIAMRE, PDE4, Karyopherin alpha 5, Importin (karyopherin)-alpha, Caprin-2, ZBTB41, **WNK1**, ZNF616, PSF, TMEM120B, NVL, NBR2, GBR2, ZFPL1, TK1, HCD2, POLA1, MCM3, FMR2, DNA ligase I, **Nidogen 1**, FATE1, MFSD1, RARbeta, RAR, **CXCR4**, HoxC10, DHRS10, Cyr61, HEATR5A, b-Myb, SYCP2L, FLJ11305, BRIP1, **HSD3B2**, Thrombospondin 4, PACE4, SAMHD1, CCDC100, Reep1, AKR1C3, OLFML2B, **ARG2**, **CYP21A2**, ITGA9, UCP2, GLYATL2, RPP40, TAPBPL, MOCOS, **STS**, TH1L, Kv4.2 channel, NOV, GABT, HPS3, FLJ10094, ATP1B2, Osteopontin, ACAD11, Sestrin 2, DYLX2, NAV3, Protein p8, CHL1, BC028528, SCFD2, CORTBP2, C5orf33, Kainate receptor, Ionotropic glutamate receptor, GRIK4, MPP7, Zyxin, Histone deacetylase class I, HDAC1
2	Digestive System Diseases	8.556E-10	6.125E-08	411	FUBP1, SYP, CRMP2, RHCG, PLA2G4C, PLA2, cPLA2, C5orf4, Stromalins 1/2, STAG1, Rwdd2a, CD72, AMP deaminase, MDGA1, DKK2, Histone H1, ENO, C5orf28, HIST1H3D, Histone H3.1, 4931414P19Rik, CRELD1, p15(PAF), MYZAP, DRIM, CD86, **Trim52**, **ACSM3**, CaMK II beta, c-Myc, DTL (hCdt2), SMP30, Neuropilin-1, PACT, YPEL2, ULBP2, GPNMB (Osteoactivin), CD101, SIA7C, KCNC2, CBS, Dynamin-3, Dynamin, SSAT, CCDC95, Rap1GAP1, C1orf141, **PPCKM**, CDC45L, Thrombospondin 3, PLEKHK1, CDC25A, CDC25, FADS2, RFC1, G-protein gamma, G-protein gamma 4, NELL2, PIAS3, C/EBP zeta, **FUS/DDIT3 fusion protein**, C/EBPgamma, C/EBP, DAX1, ERMP1, CCL2, FTL, ITGA5, GLAST1/EAAT1, GRAF, PDF, DAGK, DGKH, PLEKHG1, **PLD5**, SAT2, PAHX, Galpha(s)-specific hormone protein GPCRs, **TSH receptor**, Kv2.1, SFRS1 (SF2), Cyclophilin C, UCHL1, MCM2, **ADHFE1**, PPAPDC1B, ADA, FGF20, Galpha(i)-specific amine GPCRs, Alpha-2A adrenergic receptor, MATE1, NAV1, ULBP1, HSD3B1, ABCB6, LANCL2, **CCDC113**, R3HDM2, Whirlin, Galpha(i)-specific peptide GPCRs, Galpha(q)-specific peptide GPCRs, NR3A, WDR76, KCNH8, Neuritin1, CARD6, Sez6l, Tubulin gamma, Tubulin gamma 2, IDH2, FTDH, SFRS2 (SC-35), UGTREL7, NTN4, Ki-1/57, p21, C/EBPbeta, Cadherin 12, GDPD1, **DPP4**, LIFR, Plastin, USP45, Stathmin 3, SRp55, TMEM116, HIST1H2AK, Histone H2, HIST1H2AJ, Histone H2A, MYO5C, DALRD3, BTF, Histone H4, KIAA1958, AKR1C2, **FAM111B**, INPP, WISP-3, **PITPNC1**, XBP1, FLRT2, ANKRA, Histone H2B, Substance P, Substance P extracellular region, Neurokinin A, Neuropeptide K, Neuropeptide gamma, Na(v) I alpha, ZNFX1, Fibrillin 2, Fibrillin, MEK6(MAP2K6), **Cyclin E2**, HAS, HAS2, DEC1 (Stra13), RNase 1, **SLC35F1**, Copine-4, CCDC141, AGTR1, ZNF608, EYA2, SLC22A17, MafB, KLHDC1, DOCK10, GLB1B2,

					IL1RAP, **SERPINE2**, TFIIS, CLIP170, E4BP4, AHR, CCDC104, HIST1H2BG, ASAH, ZIM2, SIA10, RBM3, FAHD1, MAWBP, MaxiK alpha subunit, SCOT, KCTD19, **SLC43A1**, SLC25A13, U5-102 kDa, THAP2, PCDH11X, SPBC25, **EFCAB1**, SH3PXD2B, GLUT4, WARS, GINS3, MAML2, MANBA, CRMP4, IL23R, L-type Ca(II) channel, alpha 1C subunit, CACNA1 L-type, ALDOC, SGTB, PDE, ZNF577, AARS, Galpha(s)-specific peptide GPCRs, **MC2R**, SLC26A7, PEG3, ENPP1, Annexin I, TLE, ATG9A, DDX9, **ALDH1L2**, RNF213, TGF-beta 2, TGF-beta, MCM7, miR-99a-3p, miR-99a-5p, microRNA 99a, Casein kinase I, **PHYHIPL**, PLTP, COX VIb, COX VIb-2, **MALAT1**, ZNF382, Angiomotin (AMOT), Cadherin 11, Galpha(i)-specific metabotropic glutamate GPCRs, mGluR7, SLC19A2, hnRNP D-like, CYP4X1, SIAT8D, **UHRF1**, C8FW (GIG2), TMEM74, Tubulin epsilon 1, nAChR alpha, nAChR alpha-5, **DKK1**, UTP14A, RAB3IL1, JPO1, **SLFN5**, GRO-2, KCTD12, TSPYL5, Small RR subunit, **RRM2**, Pim-1, CNTN1 (F3), CYP3A7, RIT, FAM72B, GADD34, Phosphatase regulator (inhibitor), SREBP1 precursor, SREBP1 (Golgi membrane), SREBP1 (nuclear), Heme oxygenase 1, SYTL2, CENP-50, FLJ21986, ISM1, AP-3 beta subunits, Niban, IMD2, GHR, SPARCL1, CYP3A5, **LRRC17**, ENO2, Caspr1, C12orf72, PKC, PKC-eta, CACNA1 T-type, CACNG, Stargazin, GPR155, RASGRF1, ORC1L, CTH, SNX29, **Versican**, Versican proteoglycan, BY55, HZwint-1, TEX15, ALPK1, PAP39, TBL1X, FSD1, **ARHGEF2**, PGM2L1, **EGF**, ZNF25, CIA, DAF, Histone H1.5, CNR1, Galpha(i)-specific cannabis GPSRs, **Myotilin**, USP52, P4HA2, P4HA, PARC, COL15A1, LAMG1, SLC17A5, SULT2A1, F262, M2GD, ABCA1, Claspin, MR1, p56 KKIAMRE, PDE4, PDE4D, **C9orf84**, Karyopherin alpha 5, Importin (karyopherin)-alpha, Caprin-2, ZBTB41, **WNK1**, ZNF616, PSF, SC1, NVL, ASNS, MAG1, **KRT23**, GBR2, TK1, POLA1, **HIST1H2BM**, MCM3, FMR2, DNA ligase I, **Nidogen 1**, CAP-G/G2, MFSD1, Treslin, ZNF692, RARbeta, RAR, **CXCR4**, MRPS18, ESCO2, SLC7A14, Cyr61, b-Myb, MBNL3, FLJ11305, BRIP1, **PTBP2**, Caspase-4, Thrombospondin 4, TTLL3, SAMHD1, PCDH20, CCDC100, TMEM108, AKR1C3, OLFML2B, **ARG2**, **LIX1**, PSAT, ITGA9, UCP2, RPP40, MKP-3, MOCOS, FLJ20449, **STS**, NIPK, Noelin, TH1L, Semaphorin 7A, Kv4.2 channel, NOV, HPS3, FLJ10094, ATP1B2, Osteopontin, ACAD11, Sestrin 2, DYLX2, NAV3, Protein p8, CHL1, AMN1, SCFD2, CORTBP2, Kainate receptor, Ionotropic glutamate receptor, GRIK4, MPP7, ZNF330, Zyxin, Histone deacetylase class I, HDAC1, **KDELR**
					FUBP1, FAM120C, CRMP2, RHCG, PLA2, cPLA2, C5orf4, PPM1K, Stromalins 1/2, STAG1, Rwdd2a, AMP deaminase, MDGA1, DKK2, Histone H1.1, Histone H1, ENO, **CADPS**, HIST1H3D, Histone H3.1, ZCCHC4, CRELD1, MYZAP, DRIM, CD86, **ACSM3**, ALG1, CaMK II beta, PRSS16, c-Myc, DTL (hCdt2), Neuropilin-1, PACT, YPEL2, ULBP2, DOC2A, DOC2, C9orf72, FAM86A, GPNMB (Osteoactivin), HIST1H2BA, CD101, SIA7C, UHRF1BP1, UBE1L, C14orf174, CBS, MIF4GD, ACSS1, Dynamin, SSAT, Rap1GAP1, C1orf141, **PPCKM**, COL25A1, CDC45L, SLM-2, Thrombospondin 3, PLEKHK1, CDC25A,

3	Genital Diseases, Female	2.422E-07	7.198E-06	420	CDC25, G-protein gamma, G-protein gamma 4, PIAS3, C/EBP zeta, **FUS/DDIT3 fusion protein**, C/EBP, DAX1, ERMP1, C17orf45, CCL2, PLA2G1B, FTL, DGCR14, ITGA5, GLAST1/EAAT1, GRAF, PDF, DAGK, DGKH, PLEKHG1, **PLD5**, **CCPG1**, Galpha(s)-specific hormone protein GPCRs, **TSH receptor**, Kv2.1, SFRS1 (SF2), Cyclophilin C, XEDAR, UCHL1, MCM2, KLRF1, Galpha(i)-specific amine GPCRs, Alpha-2A adrenergic receptor, MATE1, NAV1, **ELOVL2**, HSD3B1, ABCB6, LANCL2, DIAC, **CCDC113**, R3HDM2, Whirlin, Galpha(i)-specific peptide GPCRs, Galpha(q)-specific peptide GPCRs, NPY1R, ALG14, NR3A, WDR76, R-Ras, NPHP3, KCNH8, Neuritin1, ARS2, Tubulin gamma, IDH2, FTDH, Ki-1/57, TMEM14B, p21, C/EBPbeta, Cadherin 12, GDPD1, **DPP4**, LIFR, Plastin, USP45, B3GT1, WIPI1, HIST1H2AK, Histone H2, HIST1H2AJ, Histone H2A, MYO5C, BTF, Histone H4, KIAA1958, **FAM111B**, **ITGA11**, FLRT2, ANKRA, CDC14b, Histone H2B, HIST1H2BN, Na(v) I alpha, ZNFX1, **Alpha-synuclein**, Fibrillin 2, Fibrillin, SLC6A6, MEK6(MAP2K6), **Cyclin E2**, HAS, DEC1 (Stra13), COQ10A, ZDBF2, Copine-4, CCDC141, AGTR1, CINAP, FUSIP1, ZNF608, EYA2, SLC22A17, DOCK10, GLB1B2, SLC9A2, **SERPINE2**, CLIP170, E4BP4, AOF1, AHR, PTCHD4, CCDC104, HIST1H2BG, ASAH, SIA10, Musculin, MaxiK alpha subunit, SCOT, KCTD19, **SLC43A1**, SLC25A13, U5-102 kDa, ZC3H6, PCDH11X, **EFCAB1**, SH3PXD2B, GLUT4, WARS, GINS3, MAML2, Peregrin, MANBA, IL23R, L-type Ca(II) channel, alpha 1C subunit, CACNA1 L-type, PDE, CLIP4, AARS, Galpha(s)-specific peptide GPCRs, SLC26A7, PEG3, ENPP1, Annexin I, TLE, TLE3, ATG9A, DDX9, **ALDH1L2**, RNF213, TGF-beta 2, TGF-beta, MCM7, miR-99a-3p, miR-99a-5p, microRNA 99a, YY2, CSNK1G1, Casein kinase I, **PHYHIPL**, PLTP, MMP-24, MRPS7, RBM14, **MALAT1**, ZNF382, Angiomotin (AMOT), Cadherin 11, Galpha(i)-specific metabotropic glutamate GPCRs, mGluR7, CHC22, NIPSNAP3B, SLC19A2, SIAT8D, **UHRF1**, C1QTNF2, TMEM74, Tubulin epsilon 1, Dynein 2, cytoplasmic, light intermediate chain, nAChR alpha, nAChR alpha-5, **DKK1**, UTP14A, RAB3IL1, JPO1, **SLFN5**, SLC25A37, TSPYL5, PPAP, Small RR subunit, **RRM2**, CNTN1 (F3), CYP3A7, GADD34, Phosphatase regulator (inhibitor), SREBP1 precursor, SREBP1 (Golgi membrane), SREBP1 (nuclear), Heme oxygenase 1, SYTL2, CENP-50, FLJ21986, AP-3 beta subunits, Niban, IMD2, GHR, CYP3A5, **LRRC17**, ENO2, Caspr1, PKC, PKC-eta, CACNA1H, CACNA1 T-type, CACNG, Stargazin, GPR155, RASGRF1, ORC1L, AGXT2L2, CTH, **Versican**, Versican proteoglycan, BY55, TEX15, TRA2A, ALPK1, PAP39, TBL1X, FSD1, SLC17A7, **ARHGEF2**, PGM2L1, **EGF**, Exportin-T, Skelemin, ZNF25, CIA, **NUDT17**, **Gdap1**, DAF, Histone H1.5, Galpha(i)-specific cannabis GPSRs, **Myotilin**, CUTC, USP52, P4HA2, P4HA, APOL1, PARC, COL15A1, PARP-6, LAMG1, SLC17A5, SULT2A1, Carmil, M2GD, ABCA1, **GnRH receptor, C/D snoRNAs**, Claspin, NHE7, TMEM55A, MR1, p56 KKIAMRE, PDE4, PDE4D, Importin (karyopherin)-alpha, Caprin-2, ZBTB41, **WNK1**, ZNF616, PSF, TMEM120B, NVL, **KRT23**, NBR2, GBR2, TK1, HCD2, DNA ligase I, **Nidogen 1**, CAP-G, CAP-G/G2, FATE1,

					MFSD1, Treslin, ZNF692, RARbeta, RAR, **CXCR4**, MRPS18, MRPS18C, HoxC10, ESCO2, SLC7A14, Cyr61, HEATR5A, b-Myb, MBNL3, FLJ11305, BRIP1, **PTBP2**, **HSD3B2**, Thrombospondin 4, TTLL3, PACE4, SAMHD1, CCDC100, Reep1, TMEM108,AKR1C3, OLFML2B, **ARG2**, **CYP21A2**, **LIX1**, TRIM73, PSAT, ITGA9, KLF15, MKP-3, SETMAR, TAPBPL, MOCOS, FLJ20449, **STS**, NIPK, TSSK2, Calcipressin 2, TH1L, Semaphorin 7A, Kv4.2 channel, NOV, GABT, HPS3,ATP1B2,SLC43A3, Osteopontin, BTN3A2, NOL9, ACAD11, Sestrin 2, DYLX2, NAV3, CHL1, **HIST1H2BB**, SCFD2, CORTBP2, POLR3GL, SCDR9, ZNF555, Kainate receptor, Ionotropic glutamate receptor, GRIK4, MPP7, Zyxin, Histone deacetylase class I, HDAC1
4	Puberty, Precocious	2.512E-07	7.327E-06	9	Galpha(s)-specific hormone protein GPCRs, Galpha(i)-specific amine GPCRs, Galpha(i)-specific peptide GPCRs, Galpha(q)-specific peptide GPCRs, NPY1R, Galpha(s)-specific peptide GPCRs, **HSD3B2**, AKR1C3, **CYP21A2**
5	Genetic Diseases, Inborn	4.396E-07	1.171E-05	141	CLCN4, CRMP2, PLA2G4C, PLA2, cPLA2, ENO, HIST1H3D, Histone H3.1, CaMK II beta, CBS, SSAT, SGK3, FADS2, G-protein gamma, NELL2, C/EBP zeta, **FUS/DDIT3 fusion protein**, C/EBP, CCL2, FTL, DGCR14, PDF, PAHX, Galpha(s)-specific hormone protein GPCRs, **TSH receptor**, XEDAR, UCHL1, MCM2, ADA, Galpha(i)-specific amine GPCRs, Alpha-2A adrenergic receptor, HSD3B1, Whirlin, Galpha(i)-specific peptide GPCRs, Galpha(q)-specific peptide GPCRs, CALB1, SFRS2 (SC-35), p21, **DPP4**, Histone H2, Histone H2A, BTF, XBP1, Substance P, Substance P extracellular region, Neurokinin A, Neuropeptide K, Neuropeptide gamma, **Alpha-synuclein**, Fibrillin 2, Fibrillin, MEK6(MAP2K6), AGTR1, SCOT, SLC25A13, GLUT4, CaMK II delta, MANBA, CRMP4, L-type Ca(II) channel, alpha 1C subunit, CACNA1 L-type, ALDOC, PDE, Galpha(s)-specific peptide GPCRs, **MC2R**, Annexin I, DDX9, TGF-beta, Tafazzin, MCM7, miR-99a-3p, miR-99a-5p, microRNA 99a, Casein kinase I, PLTP, COX VIb, COX VIb-2, COG5, Galpha(i)-specific metabotropic glutamate GPCRs, CHC22, nAChR alpha, GRO-2, PPAP, GADD34, Phosphatase regulator (inhibitor), Heme oxygenase 1, SYTL2, AP-3 beta subunits, GHR, ENO2, PKC, DGCR8, **Versican**, Versican proteoglycan, **ELOVL3**, TBL1X, **EGF**, **Gdap1**, DAF, CNR1, Galpha(i)-specific cannabis GPSRs, **Myotilin**, GAD1, COL15A1, SLC17A5, Carmil, M2GD, ABCA1, **C/D snoRNAs**, Phf11, PDE4, PWAR5, **WNK1**, NBR2, HCD2, MCM3, FMR2, RARbeta, RAR, **CXCR4**, Cyr61, BRIP1, PCDH20, Reep1, Norrin, AKR1C3, **ARG2**, **CYP21A2**, ITGA9, UCP2, **STS**, WTX, Calcipressin 2, NOV, HPS3, ATP1B2, Osteopontin, Kainate receptor, Ionotropic glutamate receptor, GRIK4, Histone deacetylase class I
6	Adenocarcinoma	4.682E-07	1.209E-05	195	FUBP1, SYP, PLA2, cPLA2, CD72, PART1, DKK2, Histone H1, ENO, HIST1H3D, Histone H3.1, p15(PAF), MYZAP, CD86, ECG2, c-Myc, DTL (hCdt2), SMP30, Neuropilin-1, GPNMB (Osteoactivin), KCNC2, CBS, Dynamin-3, Dynamin, CCDC95, CDC25A, CDC25, FADS2, G-protein gamma, G-protein gamma 4, PIAS3, C/EBP, DAX1, CCL2, FTL, GLAST1/EAAT1, PDF, DAGK, Galpha(s)-specific hormone protein GPCRs, **TSH receptor**,

					Kv2.1, XEDAR, UCHL1, RBM35A, MCM2, PPAPDC1B, ADA, Galpha(i)-specific amine GPCRs, LANCL2, Whirlin, Galpha(i)-specific peptide GPCRs, Galpha(q)-specific peptide GPCRs, NPY1R, CALB1, KCNH8, ARS2, CARD6, IDH2, FTDH, NTN4, p21, C/EBPbeta, GDPD1, **DPP4**, LIFR, Histone H2, Histone H2A, DALRD3, Histone H4, AKR1C2, **FAM111B**, **PITPNC1**, XBP1, Histone H2B, ZNFX1, Fibrillin 2, Fibrillin, **Cyclin E2**, HAS, HAS2, DEC1 (Stra13), RNase 1, Copine-4, AGTR1, SLC22A17, KLHDC1, GLB1B2, PCDH11Y, CLIP170, AHR, CCDC104, HIST1H2BG, FAHD1, MAWBP, MaxiK alpha subunit, **SLC43A1**, SLC25A13, ERV3, PCDH11X, MAML2, CRMP4, IL23R, PDE, ZNF577, Galpha(s)-specific peptide GPCRs, Annexin I, DDX9, TGF-beta 2, TGF-beta, MCM7, miR-99a-3p, miR-99a-5p, microRNA 99a, Casein kinase I, **PHYHIPL**, **MALAT1**, Angiomotin (AMOT), **UHRF1**, nAChR alpha, nAChR alpha-5, **DKK1**, UTP14A, RAB3IL1, **SLFN5**, GRO-2, TSPYL5, Small RR subunit, **RRM2**, Pim-1, RIT, Phosphatase regulator (inhibitor), SREBP1 precursor, SREBP1 (Golgi membrane), SREBP1 (nuclear), Heme oxygenase 1, Niban, SPARCL1, CYP3A5, **LRRC17**, ENO2, **MUCL1**, PKC, PKC-eta, CACNA1 T-type, **Versican**, Versican proteoglycan, HZwint-1, FSD1, **EGF**, CIA, CNR1, Galpha(i)-specific cannabis GPSRs, USP52, P4HA, PARC, COL15A1, LDB2, SULT2A1, M2GD, ABCA1, p56 KKIAMRE, PDE4, PDE4D, **C9orf84**, Importin (karyopherin)-alpha, PSF, ASNS, GBR2, TK1, FMR2, CAP-G/G2, RARbeta, RAR, **CXCR4**, HoxC10, SLC7A14, Cyr61, b-Myb, BRIP1, Thrombospondin 4, PCDH20, AKR1C3, MKP-3, MOCOS, **STS**, NOV, FLJ10094, Osteopontin, NAV3, CHL1, Kainate receptor, Ionotropic glutamate receptor, GRIK4, Histone deacetylase class I, HDAC1
7	Musculoskeletal Diseases	7.745E-07	1.848E-05	110	PLA2G4C, PLA2, cPLA2, AMP deaminase, ENO, **ENO3**, HIST1H3D, Histone H3.1, CD86, c-Myc, Neuropilin-1, CD101, CBS, Dynamin, SSAT, SKIV2L, NELL2, C/EBP zeta, **FUS/DDIT3 fusion protein**, C/EBP, CCL2, PLA2G1B, FTL, DGCR14, PDF, PAHX, Galpha(s)-specific hormone protein GPCRs, **TSH receptor**, ADA, Galpha(i)-specific amine GPCRs, Galpha(i)-specific peptide GPCRs, Galpha(q)-specific peptide GPCRs, IDH2, p21, C/EBPbeta, **DPP4**, LIFR, Histone H2, Histone H2A, BTF, WISP-3, XBP1, Histone H2B, Substance P, Substance P extracellular region, Neurokinin A, Neuropeptide K, Neuropeptide gamma, Fibrillin 2, Fibrillin, HAS, HAS2, HIST1H2BG, HIST1H2AD, MAML2, IL23R, CACNA1 L-type, PDE, Galpha(s)-specific peptide GPCRs, ENPP1, Annexin I, TGF-beta 2, TGF-beta, MMP-24, Cadherin 11, CHC22, Deoxyribonuclease II, nAChR alpha, **DKK1**, Pim-1, CNTN1 (F3), Phosphatase regulator (inhibitor), SREBP1 precursor, SREBP1 (Golgi membrane), SREBP1 (nuclear), Heme oxygenase 1, SYTL2, FLJ21986, GHR, CYP3A5, PKC, PKC-eta, DGCR8, **Versican**, Versican proteoglycan, **EGF**, DAF, CNR1, Galpha(i)-specific cannabis GPSRs, **Myotilin**, GAD1, P4HA2, P4HA, Carmil, PDE4, PDE4D, SC1, RAR, **CXCR4**, ESCO2, Cyr61, PACE4, Reep1, UCP2, WTX, Semaphorin 7A, ATP1B2, Osteopontin, Histone deacetylase class I, HDAC1

8	Obesity	2.174E-06	4.505E-05	48	PLA2, AMP deaminase, **ACSM3**, C/EBP, CCL2, PDF, DAGK, Galpha(s)-specific hormone protein GPCRs, **TSH receptor**, Galpha(i)-specific amine GPCRs, Alpha-2A adrenergic receptor, Galpha(i)-specific peptide GPCRs, Galpha(q)-specific peptide GPCRs, NPY1R, C/EBPbeta, **DPP4**, AKR1C2, XBP1, **Alpha-synuclein**, Fibrillin 2, Fibrillin, MaxiK alpha subunit, EXOD1, GLUT4, PDE, Galpha(s)-specific peptide GPCRs, ENPP1, TGF-beta, PLTP, nAChR alpha, Phosphatase regulator (inhibitor), SREBP1 precursor, SREBP1 (Golgi membrane), SREBP1 (nuclear), B4GT4, GHR, PKC, CNR1, Galpha(i)-specific cannabis GPSRs, ABCA1, PDE4, PDE4D, RARbeta, RAR, MBNL3, UCP2, NIPK, Osteopontin
9	Polycystic Ovary Syndrome	8.390E-06	1.348E-04	18	PLA2, ITGA5, Galpha(s)-specific hormone protein GPCRs, Galpha(i)-specific amine GPCRs, HSD3B1, GLUT4, ENPP1, CYP3A7, Phosphatase regulator (inhibitor), **Versican**, Versican proteoglycan, SULT2A1, **GnRH receptor**, Cyr61, AKR1C3, **CYP21A2**, **STS**, NIPK
10	Lipid Metabolism Disorders	8.618E-06	1.371E-04	25	PLA2, DAX1, CCL2, Galpha(i)-specific amine GPCRs, Galpha(i)-specific peptide GPCRs, Galpha(q)-specific peptide GPCRs, **DPP4**, AGTR1, GLUT4, Galpha(s)-specific peptide GPCRs, **MC2R**, ENPP1, PLTP, Phosphatase regulator (inhibitor), SREBP1 precursor, SREBP1 (Golgi membrane), SREBP1 (nuclear), CNR1, Galpha(i)-specific cannabis GPSRs, APOL1, ABCA1, RARbeta, RAR, UCP2, NIPK

Supplementary Table S3: **Intracellular metabolites detected by nuclear magnetic resonance spectroscopy (NMR) in human adrenal H295R cells under metformin treatment.** Data represent the relative value to the total intensity of each metabolite. Cells were grown for 48 h under growth (G) and starvation conditions (S) without and with metformin treatment (GM; SM). Data of three independent experiments are given (see also Figure 6).

ppm	**Abbreviation**	**Metabolite**	G	G	G	GM	GM	GM	S	S	S	SM	SM	SM
1.905	Ac	Acetate (Lysine, Arg)	6.24	7.62	8.58	8.37	10.64	11.27	12.06	17.34	16.23	18.46	17.17	12.24
2.285	AcA	Acetoacetic acid	0.76	0.99	0.97	1.07	1.30	1.11	0.89	0.82	1.01	1.30	1.11	0.76
average	Ala	Alanine	5.37	4.35	4.05	4.40	4.12	4.51	5.09	5.31	4.64	9.35	5.93	4.34
average	AMP	AMP	4.78	2.24	1.17	1.78	1.34	1.56	2.21	1.50	1.08	1.58	1.24	1.42
8.525	ADP	ATP, ADP	0.96	0.46	0.33	0.23	0.33	0.47	1.19	0.59	0.12	0.23	0.47	0.39
3.196	Cho	Choline	38.32	33.56	32.98	33.74	31.42	31.29	28.66	24.60	24.78	33.74	23.94	24.37
2.776	Cit	Citric acid	0.44	0.50	0.44	0.18	0.34	0.08	0.59	0.25	0.54	0.54	0.37	0.05
average	Cre	Creatine	12.31	13.47	12.01	8.90	12.49	10.50	5.18	6.84	6.31	5.59	7.27	6.70
average	Cyt	Cytidine	0.50	1.03	1.93	0.97	1.47	1.37	0.77	1.08	1.60	0.64	1.00	1.35
average	EtOH	Ethanol	1.74	0.98	1.12	1.72	1.17	1.08	2.62	1.67	2.35	3.12	1.91	2.06
average	EA	Ethanolamine	2.05	1.96	1.99	2.71	1.54	1.28	1.17	0.90	1.39	1.47	1.31	1.16
6.510	Fum	Fumarate	0.27	0.15	0.14	0.39	0.33	0.41	0.25	0.23	0.31	0.18	0.24	0.18
average	Gln	Glutamine	2.19	1.85	2.54	3.04	2.10	3.35	1.27	2.45	2.35	1.63	2.67	2.67
average	Glu	Glutamate	4.04	3.91	4.14	4.71	4.44	4.48	3.28	3.71	3.52	3.65	3.56	3.48
average	Glc	Glucose	2.88	3.64	3.94	4.28	4.26	5.52	9.21	8.38	7.98	10.09	7.49	6.47
average	GSH	Glutathion	4.22	3.61	3.52	3.94	3.44	3.46	3.35	3.27	3.09	2.54	3.01	3.40
average	Hxan	Hypoxanthine	1.07	1.77	2.12	2.65	2.50	2.43	1.60	1.59	1.94	1.62	2.02	2.20
average	Ino	Inositol	0.87	1.47	1.48	1.52	1.56	1.42	0.88	0.94	1.16	0.73	1.13	1.22
average	Ile	Isoleucine	0.77	0.69	0.73	0.71	0.65	0.72	1.29	1.27	1.35	2.72	1.48	0.95
average	Lac	Lactate	7.34	9.07	7.88	11.54	11.99	13.03	11.84	12.02	12.22	14.91	14.53	16.55
average	Leu	Leucine	6.25	5.94	5.68	5.81	5.92	5.71	11.78	11.77	12.34	26.35	13.63	6.95
average	Mal	Malic acid	0.26	0.30	0.31	0.10	0.21	0.08	0.25	0.23	0.31	0.15	0.15	0.15
2.627	Met	Methionine	0.52	0.68	0.66	0.50	0.59	0.53	0.65	0.63	0.89	1.06	0.86	0.58
average	Myo-Ino	Myo-Inositol	15.35	14.98	13.25	10.49	13.39	11.41	9.86	11.84	10.69	8.45	11.34	11.32
average	NAD	NAD	0.70	0.57	0.54	0.48	0.49	0.37	0.53	0.46	0.35	0.38	0.42	0.45
average	Niac	Niacinamide	0.38	0.17	0.32	0.55	0.28	0.52	0.21	0.18	0.26	0.30	0.27	0.39
1.242	CnHn+2	Palmitic Acid	1.88	1.75	1.68	2.09	1.88	2.35	2.65	2.25	1.96	2.10	2.35	2.63
average	PC	Phosphorylcholine	87.30	70.28	62.65	44.66	53.32	49.98	60.66	60.76	54.06	41.46	46.97	55.39
average	Phe	Phenylalanine	1.71	1.41	1.43	1.57	1.53	1.66	3.29	2.87	2.90	6.34	3.33	1.96
5.366	PEP	Phosphoenolpyruvate	0.55	0.85	1.10	0.88	1.14	1.05	0.65	0.63	0.71	0.81	0.63	0.65
average	Tau	Taurine	4.58	5.03	7.11	5.02	5.65	7.72	2.85	3.11	4.15	3.19	3.48	5.09
average	Try	Tyrosine	1.19	0.95	1.04	1.24	1.05	1.04	2.24	1.93	2.10	4.63	2.12	1.48
7.965	UDP	UDP (including all)	1.34	0.99	0.88	0.83	0.86	0.73	1.45	1.20	0.93	1.28	0.96	0.55
5.603	UDP-AcGlcA	UDP-N-AcGlcAmine	4.10	6.13	6.61	5.58	6.95	6.44	4.77	5.16	5.13	3.20	4.56	5.90
8.107	UMP	UMP	2.20	1.95	0.55	1.68	0.68	0.43	0.92	0.39	0.02	0.97	0.51	0.37
7.525	Ura	Uracil	0.29	0.09	0.24	0.10	0.00	0.24	0.34	0.34	0.63	0.85	0.47	-0.08
average	Urd	Uridine	0.33	1.11	2.63	1.09	1.93	1.80	0.90	1.34	2.43	0.78	1.36	1.58
average	Val	Valine	1.12	1.03	0.93	0.97	0.99	0.89	1.71	1.57	1.81	3.65	1.76	0.98

Supplementary Table S4: **Integrated pathway analysis of transcriptome and metabolomics data for H295R cells treated with metformin.** Analysis was performed by the IMPaLA integrated molecular pathway analysis tool on the 693 gene transcripts identified by microarray analysis and the 11 metabolites identified by NMR spectroscopy in metformin (Met) treated H295R cells. The table shows identified pathways, source of databases, number of genes and metabolites (n) and overlap between genes and metabolites. p-values and Q-values (false discovery rate) were calculated for each pathway. Metabolites are shown as PubChem ID (96, Acetoacetic acid; 311, Citric acid; 5460307, Fumarate; 33032, Glutamic acid; 790, Hypoxanthine; 91435, Lactate; 525, Malic acid; 892, Myo-Inositol; 5893, NAD; 936, Niacinamide; 24778935, Phosphocholine). Please note that the last four pathways in the table were not identified as significant pathway, but they were included anyway because of their relevance in androgen biosynthesis and mitochondrial metabolism.

**Pathway name**	**Pathway source**	**n**	**Overlapping_genes Name**	**n**	**Overlapping_metabolites ID**	**p-value**	**Q-value**
Epigenetic regulation of gene expression	Reactome	28	PHF1;HIST1H2BA;HIST1H2AJ;HIST1H2AB;HIST2H2AA3;HIST1H2BG;HIST1H2BM;HIST1H2BF;HIST1H2BI;HIST1H2BO;HIST2H2BE;HIST1H3A;HIST1H3D;HIST1H3C;HIST1H3I;HIST1H3G;HIST1H3J;HIST1H3H;HIST1H4A;HIST1H4H;HIST1H4B;HIST1H2AK;SNORA40;HIST1H2AE;UHRF1;HIST1H2BB;HDAC1;HIST1H2BJ	2	936;5893	3.00E-16	5.57E-14
Cellular responses to stress	Reactome	32	CDKN1A;HIST1H2AK;HIST1H2AJ;HIST1H2AB;HIST2H2AA3;HIST1H2BG;HIST1H4B;HIST1H2BM;HIST1H2BF;HIST1H2BI;CEBPB;HIST1H2BO;HIST2H2BE;HIST1H3A;HIST1H3D;HIST1H3C;HIST1H3I;HIST1H3G;HIST1H3J;HIST1H3H;HIST1H4A;HIST1H4H;CCNE2;CAMK2B;CAMK2D;HIST1H2BA;HIST1H1B;HIST1H2AE;HIST1H2BB;HIST1H1A;MAP2K6;HIST1H2BJ	2	936;5893	5.28E-11	3.27E-09
Signaling by Wnt	Reactome	31	MYC;HIST1H2BA;HIST1H2AJ;HIST1H2AB;HIST2H2AA3;HIST1H2BG;HIST1H2BM;HIST1H2BF;HIST1H2BI;HIST1H2BO;HIST2H2BE;HIST1H3A;HIST1H3D;HIST1H3C;HIST1H3I;HIST1H3G;HIST1H3J;HIST1H3H;HIST1H4A;HIST1H4H;HIST1H4B;TLE3;GNG2;HIST1H2AK;HIST1H2AE;HIST1H2BB;GNG4;DKK2;DKK1;HDAC1;HIST1H2BJ	2	936;5893	5.79E-11	3.48E-09
Gluconeogenesis	Reactome	5	PCK2;ENO2;ENO3;SLC25A13;ALDOC	3	311;33032;5893	1.02E-05	0.000464
Gene Expression	Reactome	59	PHF1;TCEA1;HIST1H2BJ;HIST1H2AJ;HIST1H2BM;HIST1H2AB;AARS;HIST2H2AA3;HIST1H2BG;HIST1H2BI;HIST1H2BF;PAN2;SRSF1;RARB;HIST1H2BO;ZNF577;HIST1H3A;SRSF6;RPS23;HIST1H3I;SRSF2;HIST1H3J;HIST1H3G;SNORD58A;POLR3GL;WARS;HIST1H4H;HIST1H4B;ZNF382;ZNF555;HIST1H2AK;HIST1H3D;HIST1H2BA;SNORA40;HIST1H3C;SNORD24;UHRF1;HIST1H2BB;ZNF571;ZNF616;NR0B1;SNORD38B;PRPF6;CARS2;MAML2;HIST1H2AE;DGCR8;HIST1H3H;SNRPA1;ZNF25;SNRPD1;ZNF692;HIST1H4A;HIST2H2BE;HNRNPM;HDAC1;RPL17;DHX9;PRKRA	3	936;5893;33032	1.46E-05	0.000649
Glucose metabolism	Reactome	7	PPP1R3C;ALDOC;ENO2;ENO3;PCK2;SLC25A13;PFKFB2	3	311;33032;5893	3.66E-05	0.00156

Malate-Aspartate Shuttle	SMPDB	0		3	5893;33032;525	5.12E-05	0.0021
Metabolism	Reactome	66	ARG2;SLC35D1;ENO3;FADS2;CACNA1C;PHYH;PPP1R3C;PSAT1;CYP3A7;ADHFE1;PFKFB2;ABCA1;ADRA2A;OXCT1;STS;PLA2G4C;IMPDH2;SLC25A37;ST3GAL6;IDO1;SULT2A1;IMPA1;ASAH1;INPP1;ELOVL2;ENPP1;ACSS1;CYP21A2;VCAN;UCP2;ASNS;SAT1;TAZ;SLC19A2;GLYATL2;UGT2B4;AKR1C3;PLA2G1B;ELOVL3;GPC4;HSD3B1;HSD3B2;SREBF1;IDH2;AMPD2;DSEL;RRM2;GNG4;GNG2;ADA;ALDOC;SLC25A13;ENO2;CBS;TK1;AKR1C2;PLTP;PCK2;CTH;HMOX1;SLC2A4;CA2;HSD17B10;MOCOS;TBL1X;B4GALT4	8	311;790;96;936;33032;5893;1014;892	0.000221	0.00838
SLC-mediated transmembrane transport	Reactome	12	SLC35D1;RHCG;SLC1A3;SLC6A6;SLC17A7;SLC26A7;SLC2A4;SLC9A7;SLC47A1;SLC17A5;SLC9A2;SLC43A1	5	311;790;892;33032;96	0.000251	0.00917
Glycolysis	Reactome	4	PFKFB2;ENO2;ENO3;ALDOC	2	311;5893	0.000447	0.013
Serine biosynthesis (phosphorylated route)	HumanCyc	1	PSAT1	2	33032;5893	0.0008	0.0163
Ketolysis	HumanCyc	1	OXCT1	2	96;5893	0.0008	0.0163
Butanoate metabolism - Homo sapiens (human)	KEGG	4	ACSM3;ABAT;GAD1;OXCT1	2	96;33032	0.000879	0.0173
Transmembrane transport of small molecules	Reactome	22	SLC35D1;RHCG;SLC6A6;SLC17A5;BEST1;SLC9A2;RARB;SLC26A7;SLC43A1;GNG2;SLC17A7;SLC9A7;ABCB6;SLC47A1;ATP1B2;FTL;HMOX1;GNG4;WNK1;SLC1A3;SLC2A4;SGK3	5	311;790;892;33032;96	0.00104	0.0178
Metabolism of lipids and lipoproteins	Reactome	19	SREBF1;ABCA1;OXCT1;AKR1C3;FADS2;ELOVL3;PHYH;ASAH1;AKR1C2;PLTP;ELOVL2;HSD3B1;HSD3B2;CYP21A2;TBL1X;STS;PLA2G4C;PLA2G1B;TAZ	6	311;96;5893;33032;1014;892	0.00282	0.0349
Signal Transduction	Reactome	76	RIT1;MYC;CDKN1A;HIST1H2BM;RHOQ;HIST1H2BJ;HIST1H2AJ;NRG1;HIST1H2AB;TAS2R4;HIST2H2AA3;PDE2A;THBS3;THBS4;HIST1H4B;ADRA2A;HIST1H2BF;CD55;HIST1H2BI;RARB;HIST1H2BO;HIST2H2BE;LINGO1;DKK1;HIST1H3C;TRIB3;HIST1H3I;HIST1H3G;HIST1H3J;HIST1H3H;HIST1H4A;SPP1;FGF20;ANXA1;AMOT;HIST1H4H;CD86;ARHGEF2;TLE3;ARHGAP26;HIST1H3A;PCSK6;DUSP6;AGTR1;GNG2;HIST1H2BA;GPC4;PTCH2;DNM3;AKR1C3;EGF;HIST1H2BB;CCL2;ST3GAL6;PRKCH;HIST1H2BG;DGKH;TSHR;MAML2;TAS2R31;TAC1;HIST1H2AE;PPP1R15A;TAS2R19;TAS2R20;GABBR2;GNG4;GRM7;CXCL2;DKK2;HIST1H2AK;CNTN1;HDAC1;HIST1H3D;TBL1X;NRP1	4	936;1014;5893;33032	0.00311	0.038
Steroid hormone biosynthesis - Homo sapiens (human)	KEGG	8	UGT2B4;AKR1C3;AKR1C2;CYP3A7;HSD3B1;HSD3B2;CYP21A2;STS	0		0.00369	0.043

Allopregnanolone biosynthesis	HumanCyc	2	AKR1C3;AKR1C2	1	5893	0.00373	0.0433
TCA cycle	EHMN	2	IDH2;PCK2	2	311;5893	0.0128	0.0898
Glycolysis	HumanCyc	3	ENO2;ENO3;ALDOC	1	5893	0.0227	0.13
The citric acid (TCA) cycle and respiratory electron transport	Reactome	3	ADHFE1;IDH2;UCP2	2	311;5893	0.0487	0.226
Androgen and estrogen biosynthesis and metabolism	EHMN	6	UGT2B4;CYP4X1;CYP3A7;HSD3B1;HSD3B2;STS	1	5893	0.0593	0.262
